# Early growth response 2 (EGR2) is a novel regulator of the senescence programme

**DOI:** 10.1111/acel.13318

**Published:** 2021-02-06

**Authors:** Eleanor J. Tyler, Ana Gutierrez del Arroyo, Bethany K. Hughes, Ryan Wallis, James C. Garbe, Martha R. Stampfer, Jim Koh, Robert Lowe, Michael P. Philpott, Cleo L. Bishop

**Affiliations:** ^1^ Blizard Institute Barts and The London School of Medicine and Dentistry, Queen Mary University of London London UK; ^2^ Translational Medicine & Therapeutics William Harvey Research Institute Barts and The London School of Medicine and Dentistry Queen Mary University of London London UK; ^3^ Biological Systems and Engineering Division Lawrence Berkeley National Laboratory Berkeley California USA; ^4^ Division of General Surgery Department of Surgery UCSF San Francisco California USA

**Keywords:** cellular senescence, EGR2, Ink4a, p16, replicative lifespan, senescence, transcription factor

## Abstract

Senescence, a state of stable growth arrest, plays an important role in ageing and age‐related diseases in vivo. Although the INK4/ARF locus is known to be essential for senescence programmes, the key regulators driving *p16* and *ARF* transcription remain largely underexplored. Using siRNA screening for modulators of the p16/pRB and ARF/p53/p21 pathways in deeply senescent human mammary epithelial cells (DS HMECs) and fibroblasts (DS HMFs), we identified EGR2 as a novel regulator of senescence. EGR2 expression is up‐regulated during senescence, and its ablation by siRNA in DS HMECs and HMFs transiently reverses the senescent phenotype. We demonstrate that EGR2 activates the *ARF* and *p16* promoters and directly binds to both the *ARF* and *p16* promoters. Loss of EGR2 down‐regulates p16 levels and increases the pool of p16− p21− ‘reversed’ cells in the population. Moreover, EGR2 overexpression is sufficient to induce senescence. Our data suggest that EGR2 is a direct transcriptional activator of the p16/pRB and ARF/p53/p21 pathways in senescence and a novel marker of senescence.

## Graphical abstract

Deep senescence is reversible in normal adult human fibroblasts. Loss of early growth response 2 (EGR2) reverses epithelial and fibroblast senescence. EGR2 levels are up‐regulated in multiple models of senescence and prolonged expression of EGR2 induces fibroblast senescence. Early growth response 2 (EGR2) is a novel master transcriptional regulator of the INK/ARF locus and the senescence programme.

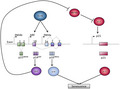

## 1 INTRODUCTION

The limited replicative capacity of cultured human cells, resulting in senescence, was first described by Hayflick and Moorhead (1961) and has since been implicated to play an important role during in vivo ageing and age‐related diseases (van Deursen, 2014). Senescence, a stable proliferative arrest, occurs in response to diverse damaging stimuli triggering up‐regulation of cyclin‐dependent kinase inhibitors (CDKIs), altered gene expression and subsequent nuclear and cellular morphological changes (Sharpless & Sherr, 2015). Two families of CDKIs, including p16^INK4A^ (p16) and p21^Cip1/Waf1^ (p21), can independently initiate senescence programmes by directly binding and inhibiting cyclin‐CDK complex phosphorylation of retinoblastoma (RB) (Dyson, 1998).

Study of *p16* regulation has revealed numerous pathways that converge to regulate *p16* and by extension the INK4/ARF locus, which also encodes *p15^INK4B^* and *p14^ARF^*/*p19^ARF^* (*ARF*) Gil & Peters, 2006; Martin et al., 2014). Importantly, ARF functions to inhibit MDM2 ubiquitination and degradation of p53, leading to up‐regulation of *p21*, a transcriptional target of p53. Thus, the INK4/ARF locus forms a pivotal link between the two key senescence initiation cascades (Zhang et al., 1998).

Epigenetic repression of the INK4/ARF locus is controlled by two polycomb repressive complexes (PRC1 and PRC2; Gil et al., 2004). In addition, individual transcription factors directly repress the *p16* promoter, including the hedgehog pathway component, GLI2 (Bishop et al., 2010), and homeobox proteins, such as HLX1, which act to recruit the PRC2 complex to the locus (Martin et al., 2013). Similarly, T‐box proteins, TBX2 and TBX3, directly repress the *ARF* promoter (Brummelkamp et al., 2002; Jacobs et al., 2000).

Although it is well established that ETS1 mediates *p16* induction in fibroblasts by the RAS/RAF/MEK cascade during oncogenic signalling, leading to oncogene‐induced senescence (Serrano et al., 1997), the upstream pathways activating the INK4/ARF locus in epithelial and fibroblast senescence are not well understood. To date, overexpression of the homeobox protein, MEOX2, has been identified to induce senescence in keratinocytes and fibroblasts by directly binding to and activating the *p16* promoter (Irelan et al., 2009), and overexpression of E2F1 induces senescence in fibroblasts via increased *ARF* expression (Dimri et al., 2000). Depending on the cellular context, β‐catenin can directly activate (Wassermann et al., 2009) or repress *p16* (Delmas et al., 2007), whilst FOXO proteins can directly activate *p15* and *ARF* (Katayama et al., 2008) or repress *p16* (Yalcin et al., 2008).

Furthermore, recent evidence has suggested that senescence is a multi‐step, dynamic process throughout which the senescent phenotype evolves (Kim et al., 2013). Deep senescence (DS) takes over 7–10 days to develop post‐senescence induction. For example, in epithelial cells, it is defined when cultures at p16‐dependent stasis undergo no further expansion upon at least two serial passages (Lowe et al., 2015, Methods). In fibroblasts, it is further characterised by additional markers of senescence, most notably the senescence‐associated secretory phenotype (SASP) (Coppé et al., 2008; Rodier et al., 2009), accompanied by elevated reactive oxygen species (ROS) levels (Lowe et al., 2015; Passos et al., 2010) and a loss of lamin B1 (Freund et al., 2012). Despite our growing understanding of the elaboration of the senescent state, there is a lack of knowledge of the key regulatory pathways upstream of the p16/pRB and ARF/p53/p21 pathways in DS.

**FIGURE 1:**
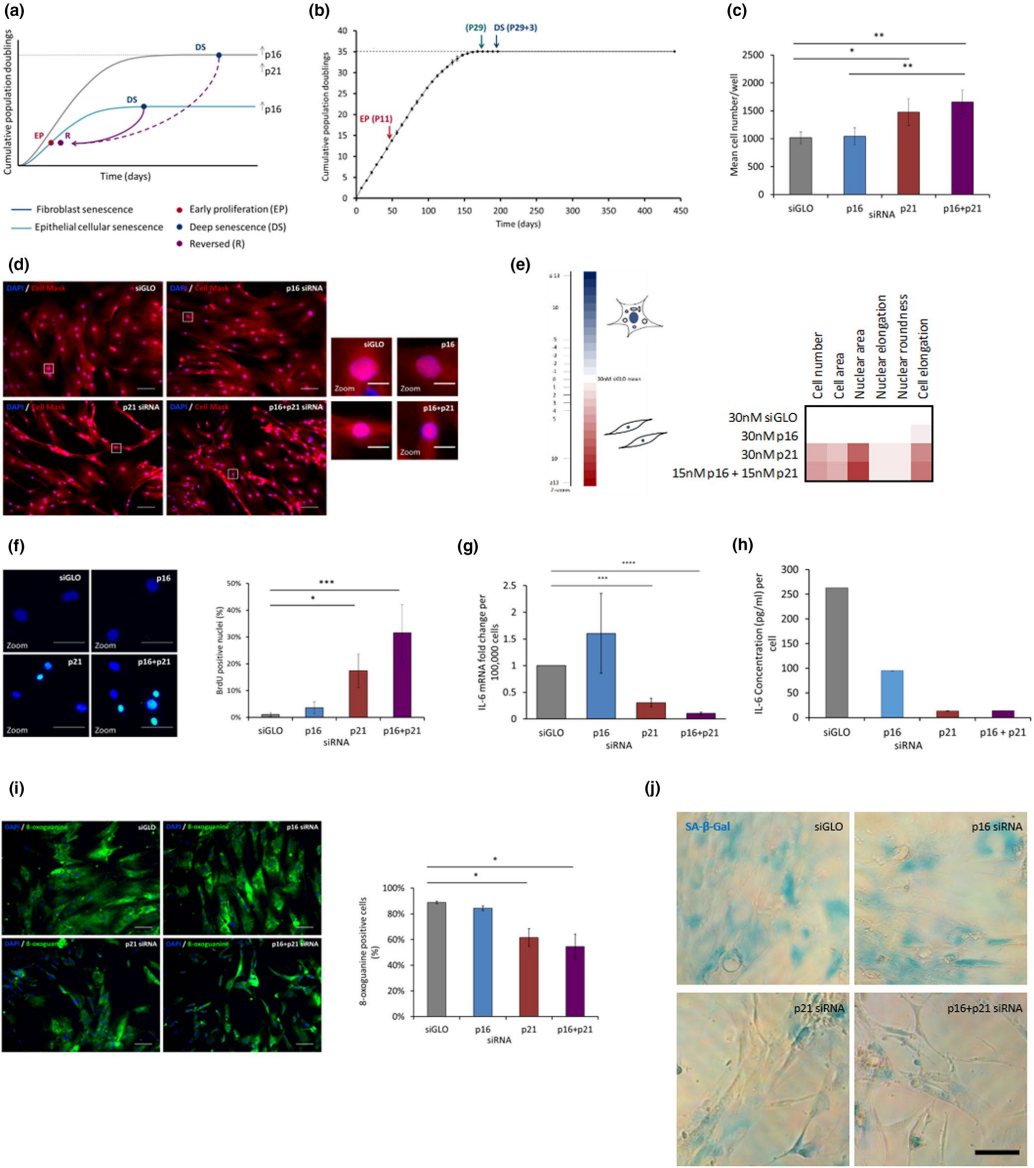
Deep senescence (DS) in primary adult human mammary fibroblasts is reversible. (a) Schematic illustrating epithelial and fibroblast senescence and the DS reversal strategy. (b) Early proliferating (EP) fibroblasts at P11 were serially passaged until they reached senescence at P29. Deeply senescent (DS) fibroblasts were defined as a population which did not expand when kept in culture for 3 weeks post‐senescence (P29 + 3). No expansion was observed in DS fibroblasts kept in culture for a further 130 days. *N* = 1 between P4 and P6; *N* = 2 or more between P7 and P29 + 3; *N* = 1 P29 + 3 + 130 days. Error bars = SD of at least two independent experiments. (c–j) DS HMFs were forward transfected with 30 nM control siRNA (siGLO), 30 nM *p16* siRNA (p16), 30 nM *p21* siRNA (p21) or 15 nM *p16* siRNA together with 15 nM *p21* siRNA (p16+p21) and fixed 5 days post‐transfection (b–e, h–i), harvested for RTqPCR at 72 h post‐transfection (f) and conditioned medium collected 5 days post‐transfection (g). (c) Bar chart showing mean cell number/well. *p < 0.05, **p < 0.01. Error bars, SD from four independent experiments, each performed with three replicates. (d) DS HMFs stained with DAPI (blue) and Cell Mask (red). Size bar 100 µm. Right panel = digital zoom. Size bar 20 µm. (e) Multi‐parameter analysis of cellular and nuclear morphological measures. Colour coding used to illustrate the number of Z scores of the experimental siRNA value from the siGLO mean. (f) DS HMFs stained with DAPI (blue) and anti‐BrdU (green). Size bar 50 µm. Bar chart showing mean BrdU positive nuclei for each condition. *p < 0.05, ***p < 0.001. Error bars, SD from three independent experiments, each performed with three replicates. (g) RTqPCR analysis of mRNA levels of *IL*‐*6* in DS HMFs ***p < 0.001, ****p < 0.0001. Error bars, SD from two independent experiments, each performed with two replicates. (h) Representative ELISA of secreted IL‐6 levels in DS HMFs. (i) DS HMFs stained with DAPI (blue) and anti‐8‐oxoguanine (green). Size bar 100 µm. Bar chart depicting mean 8‐oxoguanine positive cells for each condition. *p < 0.05. Error bars, SD from two independent experiments, each performed with three replicates. (j) Representative images of DS HMFs stained for senescence‐associated beta‐galactosidase (SA‐β‐Gal) activity (blue). Size bar 50 µm

We have previously demonstrated that DS is reversible in p16‐positive primary adult human mammary epithelial cells (HMECs) using *p16* siRNA transfection (Lowe et al., 2015). Of relevance, p16‐dependent epithelial senescence is independent of ARF/p53/p21 pathway activation (Garbe et al., 2009), whereas senescence in primary adult human fibroblasts engages both the ARF/p53/p21 and p16/pRB pathways (Alcorta et al., 1996; Figure 1a). We took note of previous work in human neonatal foreskin fibroblasts (HCA2) which demonstrated that p53 knockdown in senescence reinitiates DNA synthesis but with limited proliferation (Gire & Wynford‐Thomas, 1998), and subsequent findings that p53 or pRB inactivation in neonatal foreskin fibroblasts (BJ), with low levels of p16, reversed senescence (Beauséjour et al., 2003). However, p53 inactivation or p16 shRNA knockdown followed by p53 inactivation in foetal lung WI38 fibroblasts, with higher levels of p16, did not reverse senescence, leading the authors to suggest that activation of the p16/pRB pathway may provide a dominant second barrier to senescence reversal (Beauséjour et al., 2003).

Here, we show that DS in primary adult human fibroblasts with high p16 levels can be reversed using transfection of *p16* siRNA in combination with *p21* siRNA. Subsequently, we perform siRNA screens in DS HMECs and human mammary fibroblasts (HMFs) in order to further understand the key regulators upstream of the p16/pRB and ARF/p53/p21 pathways which drive senescence. In this study, we present evidence that early growth response 2 (EGR2) acts as a transcriptional activator of *p16* and *ARF* in senescence and is a novel marker of senescence.

## 2 RESULTS

### 2.1 Reversal of deep senescence in fibroblasts

Current literature suggests that senescence is a dynamic process and that fibroblasts in ‘light’ senescence (with low p16 levels) can be reversed, whereas DS fibroblasts (with high p16 levels) have entered a distinct, irreversible state (Beauséjour et al., 2003). As such, we began by asking whether fibroblast DS (with high p16 and p21 levels) is truly irreversible. Building on previous work in which we have reversed DS in p16‐positive DS HMECs (Lowe et al., 2015), we hypothesised that transient knockdown using previously validated *p16* (Bishop et al., 2010) together with *p21* (Borgdorff et al., 2010) siRNAs in DS fibroblasts would induce a ‘reversed phenotype’ as characterised by a panel of senescence markers (Figure 1a).

To investigate this hypothesis, we employed senescent HMF and human dermal fibroblasts (HDFs) that had been serially passaged to senescence and cultured for a further 21 days to ensure a deeply senescent state with high p16 and p21 levels (Figure 1b, Figure S1, Methods) and developed an efficient protocol to introduce siRNA into these classically hard to transfect cells (Methods). Subsequently, we depleted *p16* and/or *p21* mRNA in DS HMFs or HDFs with potent siRNAs (Figure S2A,B) and assessed the impact on numerous cellular and molecular markers classically associated with senescence in comparison to DS cells transfected with siGLO (a negative control targeting cyclophilin B (PPIB); ‘DS + siGLO’). Whilst depletion of *p16* with siRNA in DS HMFs (‘DS + p16 siRNA’) did not significantly alter the arrested phenotype or cellular and molecular markers of senescence, *p21* depletion (‘DS + p21 siRNA’) significantly increased cell number and modulated some features of senescence morphology towards an early proliferating (EP) phenotype, namely, significantly decreased cell area, nuclear area and nuclear elongation; and significantly increased nuclear roundness and cell elongation (Figure 1c‐e). Strikingly, depletion of both *p16* and *p21* in DS HMFs and HDFs (‘DS + p16 + *p21* siRNA’) stimulated a stronger reversion to an EP morphology as characterised by multiple cellular and molecular markers (Figure 1c–e, Figure S3). Using a panel of established senescence markers, we sought to explore further the consequences of *p16* and *p21* knockdown. Quantification of proliferation using 5‐bromo‐2’‐deoxyuridine (BrdU) incorporation confirmed the significantly increased cycling activity of ‘DS + p16 + *p21* siRNA’ HMFs compared to ‘DS + siGLO’ HMFs (Figure 1f). Interestingly, the percentage of BrdU positive cells in ‘DS + p16 + *p21* siRNA’ HMFs was higher even than that observed in EP HMFs (Figure S1D), indicating that a greater proportion of the ‘DS + p16 + *p21* siRNA’ HMFs progress through S phase during the 16‐h BrdU pulse than the EP HMFs. In agreement with the reversed phenotype, ‘DS + p16 + *p21* siRNA’ HMFs also displayed down‐regulation of the SASP proinflammatory signature in comparison to ‘DS + siGLO’ HMFs, as illustrated by significantly decreased expression of the cytokine *IL*‐*6* (Figure 1g) and decreased IL‐6 secretion (Figure 1h). In line with the literature, IL‐8 expression and secretion were also investigated but found not to be a feature of the SASP in DS HMFs (data not shown; Coppé et al., 2008). We also measured levels of 8‐oxoguanine, a marker of reactive oxygen species and oxidative damage, and found a significant decrease in the ‘DS + p16 + *p21* siRNA’ population compared to ‘DS + siGLO’ HMFs (Figure 1i). Furthermore, investigation of senescence‐associated beta‐galactosidase (SA‐β‐Gal) activity in DS HMFs following transfection, suggested a potential decrease in SA‐β‐Gal activity in ‘DS + p16 + *p21* siRNA’ HMFs compared to ‘DS + siGLO’ HMFs (Figure 1j). Together, our data indicate that senescence appears to be transiently reversed in the ‘DS + p16 + *p21* siRNA’ HMFs.

### 2.2 siRNA screening reveals novel regulators of senescence

**FIGURE 2:**
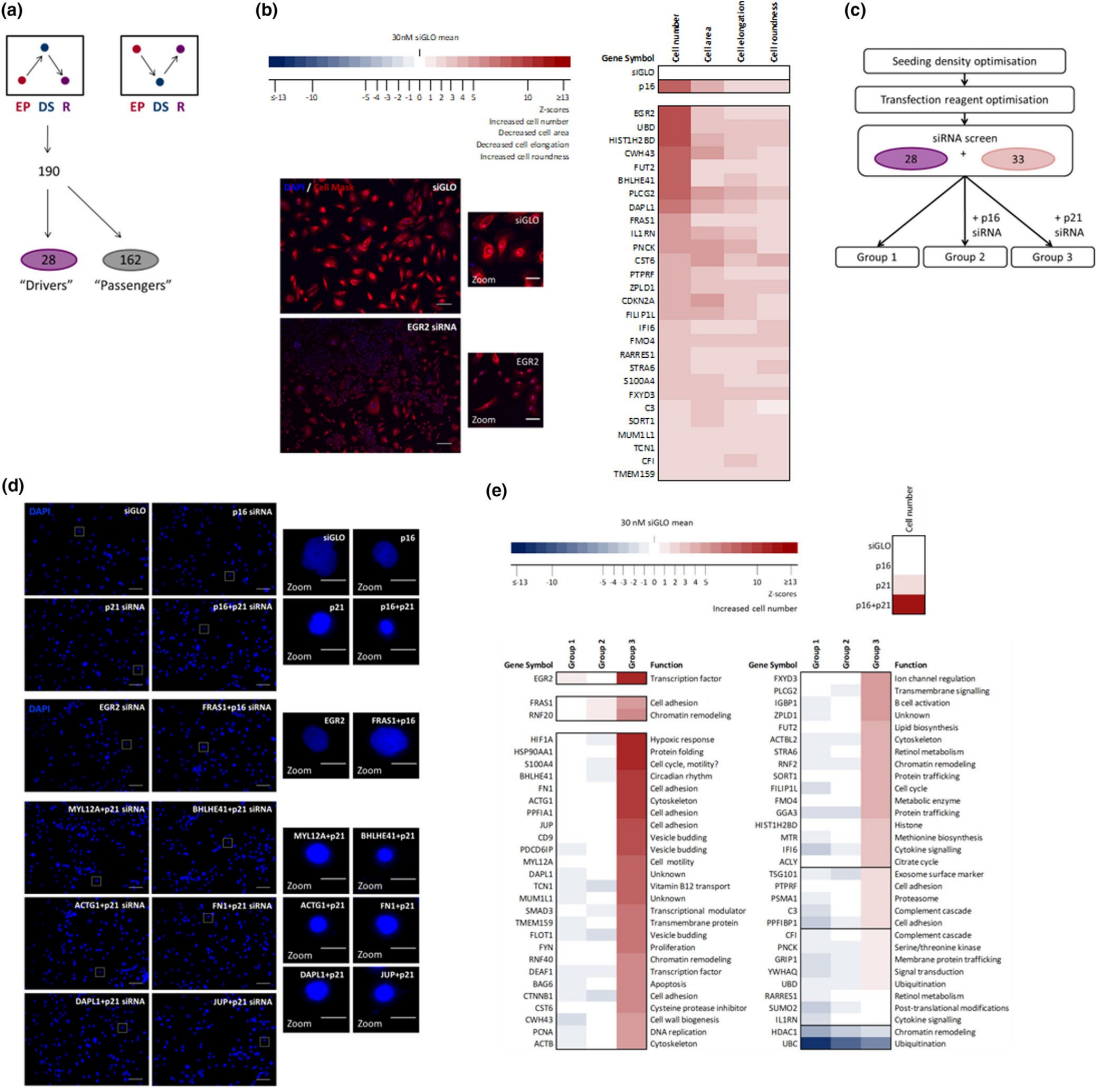
High‐content screening for regulators of senescence. (a) Schematic illustrating mRNA microarray data which identified top 190 genes with increased expression in the deeply senescent (DS, blue) versus the early proliferating (EP, red) and reversed (R, purple) HMECs. (b) Results of DS HMEC screen performed twice, in triplicate. Colour coding used to illustrate the number of Z scores of the experimental siRNA value from the siGLO mean. Heatmap of Z scores for cell number, cell area, cell elongation and cell roundness following transfection of DS HMECs. DS HMECs stained with DAPI (blue) and Cell Mask (red) following transfection with control siRNA (siGLO) or siRNAs targeting representative hit (*EGR2*). Size bar 100 µm. Right panels = digital zoom. Size bar 100 µm. (c) Schematic illustrating the experimental design of the siRNA screen. DS HMFs were forward transfected with the 60 target siRNAs in three conditions: 30 nM siRNA individually (Group 1); 15 nM siRNA in combination with 15 nM *p16* siRNA (Group 2); and 15 nM siRNA in combination with 15 nM *p21* siRNA (Group 3). (d) DS HMFs stained with DAPI (blue) following transfection with control siRNAs (siGLO, *p16*, *p21*, *p16* + *p21*) or siRNAs targeting representative hits (*EGR2*, *MYL12A*, *BHLHE41*, *ACTG1*, *FN1*, *DAPL1*, *JUP*). Size bar 100 µm. Right panels = digital zoom. Size bar 20 µm. (e) DS HMF screen performed twice, in triplicate. Colour coding used to illustrate the number of Z scores of the experimental siRNA value from the siGLO mean. Heatmap of Z scores for cell number following transfection of DS HMFs with Group 1, Group 2 or Group 3 siRNAs. A brief function is assigned to each siRNA

We next sought to identify novel genes that regulate the senescent phenotype. Initially, we interrogated our previously published gene expression datasets to identify genes whose expression was significantly up‐regulated in HMEC DS relative to EP HMECs and down‐regulated following *p16* siRNA knockdown (Figure 2a; Lowe et al., 2015; GEO: GSE58035, *q* < 0.05). In order to distinguish between the genes driving senescence and downstream ‘passenger’ genes, a siRNA screen of the top 190 genes was performed in DS HMECs (Table S1). Each gene was targeted by a pool of three siRNAs (30 nM Ambion). To determine the effect on a panel of senescence markers for each of the 190 siRNAs, the siGLO transfected control provided a baseline for Z score generation. Using high‐content analysis, 28 siRNAs (14.7%) were identified to strongly induce reversal in the DS HMECs as defined by an increase in cell number and the loss of a panel of senescence markers (i.e. mimicking the HMEC phenotype generated by *p16* siRNA). Accordingly, these 28 genes were classified as potential regulators of senescence (Figure 2b).

To further investigate the relationships between these potential 28 regulators of senescence, we constructed a protein interaction map. Briefly, these 28 genes were probed for protein interactors using the BioGRID database (Figure S4). Using Panther, KEGG pathways and Gene Ontology (GO) bioinformatics tools, 61 genes emerged (the 28 previously identified regulators, which includes p16 and 33 protein interactors) which grouped into six functional categories: immune response; cell adhesion/cytoskeleton; metabolism; transcription; growth/proliferation; and protein/vesicle trafficking (Figure S5).

We next asked whether the siRNA hits that emerged from the initial HMEC screen could also play a role in senescence in DS HMFs using this extended protein interaction network. As DS HMF reversal was found to require siRNA knockdown of both *p16* and *p21*, we hypothesised that the regulators identified in the DS HMEC screen may additionally require knockdown of either the p16/pRB or the ARF/p53/p21 pathway to induce reversal in the DS HMFs. Accordingly, DS HMFs were screened with 60 target siRNAs (27 regulators, excluding p16 and 33 interactors) in three conditions: 30 nM siRNA individually (Group 1); 15 nM siRNA in combination with 15 nM *p16* siRNA (Group 2); or 15 nM siRNA in combination with 15 nM *p21* siRNA (Group 3) (Figure 2c).

Using the same approach as described for the DS HMEC siRNA screen, a hit list was generated for each of the three conditions (Groups 1, 2 and 3) (Figure 2d,e). One siRNA transfected individually (Group 1) was defined as a hit, namely early growth response 2 (*EGR2*), a transcription factor involved in several cellular processes including cell cycle and proliferation (Parkinson et al., 2004; Srinivasan et al., 2012). Two siRNAs in combination with *p16* siRNA (Group 2), fraser extracellular matrix complex subunit 1 (*FRAS1*) and ring protein 20 (*RNF20*), an E3 ubiquitin ligase, were defined as hits (Figure 2d–e). Finally, 45 of the 60 siRNAs in combination with *p21* siRNA (Group 3) were defined as hits. Strikingly, eight of these 45 siRNAs induced an increase in cell number similar to the ‘DS + p16 + *p21* siRNA’ DS HMF control, including *EGR2* and *S100A4* siRNA. As the 28 regulator siRNAs in the screen were identified as hits for senescence reversal in p16‐dependent DS HMECs, it is perhaps unsurprising that 21 of these siRNAs were identified as hits requiring additional knockdown of the ARF/p53/p21 pathway to reverse senescence in DS HMFs. Furthermore, 24 of the 33 interactors investigated in this screen were also identified as Group 3 hits, highlighting the utility of the bioinformatics approach.

The top candidates from Group 1 (*EGR2*) and Group 2 (*FRAS1*), together with an additional 12 candidates from Group 3 were selected for further investigation (*HIF1A*, *HSP90AA1*, *S100A4*, *BHLHE41*, *FN1*, *ACTG1*, *PPFIA1*, *JUP*, *CD9*, *PDCD6IP*, *MYL12A* and *DAPL1*). We performed a more detailed, independent screen with these 14 siRNAs using multi‐parameter analysis of senescence‐associated morphological markers with four conditions: 30 nM siRNA individually (Group 1), 15 nM siRNA in combination with 15 nM *p16* siRNA (Group 2); or 15 nM siRNA in combination with 15 nM *p21* siRNA (Group 3) (Figure S6). In addition, the impact of an increased individual siRNA dose (60 nM, Group 1B) was performed to identify the most potent reversed phenotype (Figure S6).

Strikingly, 11 of the 14 siRNAs transfected individually significantly decreased cell area in a dose‐dependent manner (Group 1, Group 1B; Figure S7). Of these, six siRNAs transfected individually also significantly decreased nuclear area in a dose‐dependent manner (Group 1, Group 1B) and *EGR2* was the only siRNA transfected individually (Group 1, Group 1B) to also significantly increase cell elongation in a dose‐dependent manner. As such, EGR2 was the only siRNA that did not require knockdown of *p16* and *p21* to significantly increase cell number (Figure 2) and significantly alter three senescence‐associated morphologies towards a reversed phenotype in a dose‐dependent manner (Figure S7). Taken together, these data suggest that EGR2 may be acting upstream of *p16* in epithelial DS and *p16* and *p21* in fibroblast DS. To our knowledge, no direct relationship between EGR2 and senescence has previously been described, and thus, we sought to explore this finding in more detail.

### 2.3 EGR2 is a novel regulator of senescence

**FIGURE 3:**
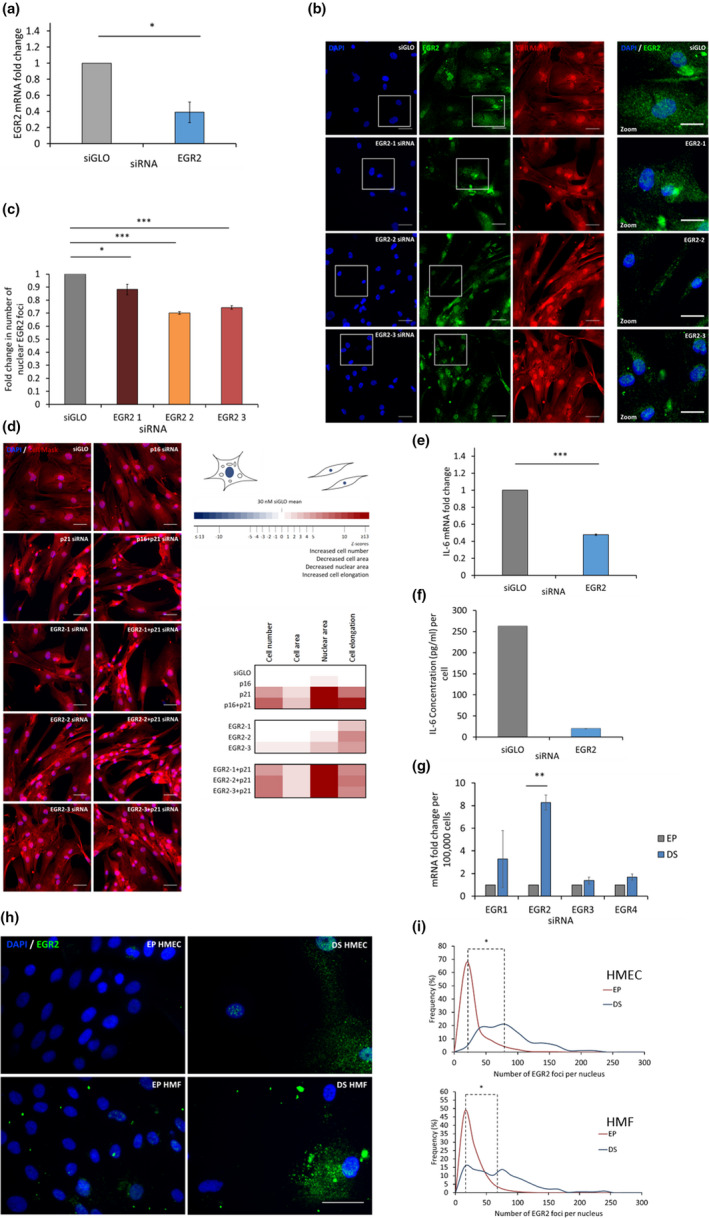
EGR2 knockdown in vitro reverses senescence‐associated morphologies and down‐regulates SASP component, IL‐6 and EGR2 protein levels increase in deep epithelial and fibroblast senescence in vitro. (a) RTqPCR analysis of mRNA levels of *EGR2* in DS HMFs following siGLO or *EGR2* knockdown. **p < 0.01. Error bars, SD from two independent experiments, each performed with two replicates. (b) Representative immunofluorescence images of DS HMFs stained with DAPI (blue), EGR2 (green) and Cell Mask (red) following transfection with siGLO or deconvoluted *EGR2* siRNAs at 5 days post‐transfection. Size bar 50 µm. Right panel = digital zoom. Size bar 30 µm. (c) Bar chart depicting median EGR2 nuclear foci following siGLO or *EGR2* siRNA knockdown. *p < 0.05, **p < 0.01, ***p < 0.001. Error bars, SD from two independent experiments, each performed with three replicates. (d) Representative immunofluorescence images of DS HMFs stained with DAPI (blue) and Cell Mask (red) following transfection with control siRNAs (siGLO, *p16*, *p21*, *p16* + *p21*) or deconvoluted siRNAs targeting *EGR2*. Size bar 50 µm. Heatmap depicting Z scores for phenotypic validation following *EGR2* siRNA pool deconvolution in DS HMFs. Two independent experiments were performed, each in triplicate. (e) RTqPCR analysis of mRNA levels of *IL*‐*6* in DS HMFs following siGLO or *EGR2* knockdown. ***p < 0.001. Error bars = SD from two independent experiments, each performed with two replicates. (f) Representative ELISA of secreted IL‐6 levels in DS HMFs following transfection with control siRNA (30 nM siGLO) or 30 nM *EGR2* siRNA (EGR2). (g) RTqPCR analysis of mRNA levels of EGR family members (*EGR1*, *EGR2*, *EGR3*, *EGR4*) in EP and DS HMFs. **p < 0.01. Error bars, SD from two independent experiments, each performed with two replicates. (h) EP and DS HMECs and HMFs stained with DAPI (blue) and EGR2 (green). Size bar 50 µm. (i) Frequency distributions of EGR2 nuclear foci in EP and DS HMECs and HMFs. *p < 0.05. Two independent experiments, each containing three technical repeats were performed

As EGR2 was identified as the top hit for reversal in both the DS HMEC and HMF screens, we next wanted to explore the role of EGR2 in senescence. First, we validated mRNA knockdown for the *EGR2* siRNA pool in DS HMFs (Figure 3a) and subsequently deconvoluted the *EGR2* siRNA pool (*EGR2 1*, *2* and *3*) to determine the efficacy of each individual siRNA targeting *EGR2*. ‘*EGR2 1*’ siRNA was the least potent (Figure S8), which was subsequently reflected in the phenotype (Figure 3b–d). Using multi‐parameter phenotypic analysis to control for off‐target effects, we identified ‘*EGR2 3*’ siRNA as the most potent siRNA transfected individually (Figure S8) which significantly increased cell number and significantly reversed cell area, nuclear area and cell elongation (Figure 3b–d). ‘*EGR2 2*’, the second most potent siRNA transfected individually (Figure S8), produced a modest increase in cell number and significantly reversed nuclear area and cell elongation (Figure 3b–d). Finally, the least potent siRNA, ‘*EGR2 1*’, only significantly reversed cell elongation compared to the DS + siGLO control (Figure 3d). Further characterisation of the changes to the senescence phenotype following ablation of the *EGR2* in DS HMFs revealed a significant down‐regulation of the SASP factor, *IL*‐*6*, at the transcript level (Figure 3e) and at the secreted protein level (Figure 3f). As mentioned previously, IL‐8 is known not to be a feature of the DS HMF SASP (data not shown, Coppé et al., 2008).

It is important to note that the human genome encodes four EGR transcription factors, EGR1‐4, that share three highly homologous DNA binding zinc finger domains that can bind to the same GC‐rich consensus DNA binding motif (Beckmann & Wilce, 1997). In addition, a role for EGR1 has previously been implicated in RAF‐induced oncogene‐induced senescence (OIS) of human BJ fibroblasts (Carvalho et al., 2019) and replicative senescence (RS) of murine embryonic fibroblasts (Krones‐Herzig et al., 2003). As such, we wanted to investigate the expression of EGR family members in HMEC epithelial senescence and HMF senescence. EGR2 was the only member of the EGR family with significantly increased gene expression in DS compared to EP HMECs, and EGR2 was the only member of the EGR family whose gene expression significantly decreased in reversed HMECs (GEO: GSE58035). Furthermore, investigation of EGR family member expression levels in EP and DS HMFs revealed a significant increase in EGR2, but not EGR1, EGR3 or EGR4 expression levels (Figure 3g). Collectively, these data suggest that EGR2 might be the key EGR family member acting to regulate senescence in HMECs and HMFs.

Subsequently, to further explore whether EGR2 activity and regulation is conserved across multiple senescence models and occurs in vivo in human tissues, we performed data mining of existing GEO datasets for HDF RS, bleomycin‐induced stress‐induced premature senescence (SIPS) in BJ foreskin fibroblasts and RAS oncogene‐induced senescence (OIS) in WI38 lung fibroblasts (Martínez‐Zamudio et al., 2020) in vitro, as well as human skin and whole‐blood with age in vivo (STAR Methods). The abundance of EGR2 increased during senescence across all three senescence models (Figure S9A, *p* < 0.05). Importantly, EGR2 expression increased in vivo in aged human skin. In addition, a recent whole‐blood gene expression meta‐analysis looking at over 7000 human samples showed that EGR2 expression significantly increases with age (Figure S9, *p* < 0.01, Peters et al., 2015). Thus, increased EGR2 expression appears to be a feature of both in vitro senescence and in vivo ageing signatures.

EGR2 possesses a nuclear localisation signal and functions to regulate gene transcription within the nucleus, thus we hypothesised that functional EGR2 would be localised within the nucleus during senescence. Immunofluorescence staining in EP and DS HMECs revealed a significant increase of nuclear EGR2 foci in DS HMECs compared to the EP population and in DS HMFs compared to EP HMFs (Figure 3h–i). Further investigation of EGR2 levels in a third model of senescence, oncogene‐induced senescence (OIS) in IMR90 lung fibroblasts (Figure S9B), identified a significant increase in nuclear EGR2 foci in OIS fibroblasts compared to the vector control (Figure S9C). These findings support our previous mining of mRNA datasets and show that an increase in EGR2 is also observed at the protein level with the expected subcellular localisation (Figure 3h–i), thus identifying EGR2 as a novel marker of senescence in both DS HMECs, HMFs and OIS IMR90 fibroblasts.

Finally, to explore the potential mechanisms through which EGR2 may be driving senescence and identify a panel of genes that might be regulated by EGR2 during senescence, we asked if genes identified to be up‐regulated in senescence in the HMEC gene expression array were enriched for the previously published EGR2 consensus binding sequences (ACGCCCACGCA; Jolma et al., 2013; Mathelier et al., 2016) compared to randomly sampled background gene sets (Figure S10A–C). Interestingly, there was a small but significant enrichment for EGR2 binding sites at the promoters of genes up‐regulated in HMEC DS. Furthermore, ten of these genes were identified as hits for senescence reversal in the DS HMEC screen, including *p16*, and nine of these were also identified as hits in the HMF siRNA screen, including the top hit *S100A4*, suggesting that EGR2 may act as a senescence regulator by activating the expression of these genes.

### 2.4 EGR2 regulates senescence via the p16/pRB and ARF/p53/p21 pathways

**FIGURE 4:**
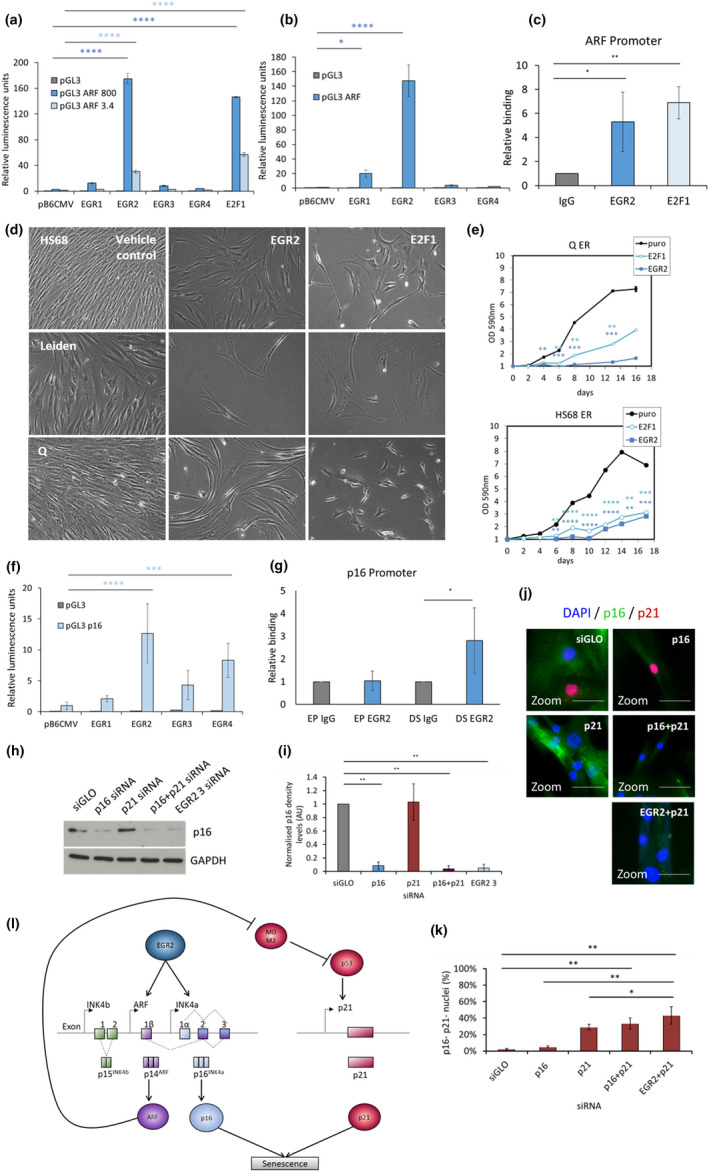
EGR2 directly binds to *ARF* and up‐regulates p16 and ARF which is sufficient to induce proliferation arrest. (a–b) Mean luciferase values for activation of pGL3 luciferase reporter constructs harbouring either the promoter sequence up to 800 bp or 3.4 kb upstream of the transcriptional start site of *ARF* (pGL3 ARF 800 or ARF 3.4, respectively) following co‐transfection of U2OS cells with expression vectors encoding each of the EGR family members (EGR1‐4) or E2F1 (a positive control). Error bars, SD from two experiments. (c) Chromatin immunoprecipitation (ChIP) showing relative levels of EGR2 or E2F1 binding to the ARF promoter in quiescent Kit225 human T‐lymphocytes or Kit225 lymphocytes following IL‐2 activation. *p < 0.05, **p < 0.01. Error bars = SD from three independent experiments. (d) Representative images of Hs68, p16^−/−^ Leiden or p16^+/−^ Q cells following infection with retroviral particles expressing EGR2 cDNA and selection on puromycin. Images taken at the same magnification. (e) Hs68 fibroblasts or p16^+/−^ Q cells were infected with retroviral particles expressing the indicated cDNAs, selected on puromycin and assessed for proliferative capacity by periodic trypsinisation and cell counting. **p < 0.01, ***p < 0.001, ****p < 0.0001. Error bars = SD from three experiments. (f) Mean luciferase values for activation of pGL3 luciferase reporter constructs harbouring *p16* promoter sequence (pGL3 p16) following co‐transfection of U2OS cells with expression vectors encoding each of the EGR family members (EGR1‐4) or E2F1 (a positive control). Error bars, SD from six experiments. (g) ChIP showing relative levels of EGR2 binding to the p16 promoter in EP and DS HMFs. *p < 0.05. Error bars = SD from four independent experiments. (h) Representative Western blots depicting p16 levels in DS HMFs following transfection with control siRNA (siGLO), *p16* siRNA (p16), *p21* siRNA (p21), *p16* siRNA together with *p21* siRNA (p16 + p21) or individual *EGR2* siRNA 3 (EGR2 3). Lysates were probed for mouse anti‐p16 (JC8) and the rabbit anti‐GAPDH antibody was used as a loading control. (i) Densitometry analysis of p16 levels in transfected DS HMFs. Analysis was performed using ImageJ software. Bars denote mean density levels. One‐way ANOVA and Dunnett's test **p < 0.01. *N* = 2 throughout. Error bars = SD normalised to siGLO siRNA of two independent experiments. (j) Representative immunofluorescence images of DS HMFs stained with DAPI (blue), p16 (green) and p21 (red) following transfection with control siRNA (siGLO), *p16* siRNA (p16), *p21* siRNA (p21), *p16* siRNA together with *p21* siRNA (p16 + p21) or *EGR2* siRNA together with *p21* siRNA (EGR2 + p21). Digital zoom. Size bar 50 µm. (k) Bar chart depicting mean p16 and p21 negative (p16− p21−) nuclei for transfected DS HMFs. *p < 0.05, **p < 0.01. Error bars, SD from two independent experiments, each performed with three replicates. (l) Schematic summarising the proposed relationship between EGR2 (dark blue), ARF (purple), p16 (light blue), MDM2, p53 and p21 (red) in senescence.

Although previous work has identified EGR2 binding to the *p21* promoter (Srinivasan et al., 2012; Zheng et al., 2013), no investigation has yet been performed on other pathways of senescence (Figure 4j). Further examination of the INK4/ARF locus revealed previously unreported hypothetical EGR2 binding sites (ACGCCCACGCA; Jolma et al., 2013; Mathelier et al., 2016) in the *p16*, *p15* and *ARF* promoter regions, indicating a potential for EGR2 to bind to and regulate expression of *p16*, *p15* and *ARF*. As *p15* was found not to be expressed in DS HMFs (Figure S10D), we explored the potential action of EGR2 on the *p16* and *ARF* promoters. To this end, we first investigated activation of the *ARF* promoter using transiently co‐transfected U2OS cells with an expression vector encoding one of each of the four members of the EGR family or E2F1, a transcription factor known to directly up‐regulate ARF which acts as a positive control (Dimri et al., 2000), together with pGL3 luciferase reporter constructs harbouring either the promoter sequence 800 bp or 3.4 kb upstream of the transcriptional start site of *ARF* (pGL3 ARF 800 or plGL3 ARF 3.4, respectively, Figure 4a,b). Cells transfected with the pGL3 ARF 800 or with the complete ARF promoter, pGL3 ARF 3.4, displayed a significant increase in luciferase activity following transfection with the EGR2 expression vector or E2F1 positive control, but not EGR1, EGR3 or EGR4 expression vectors, thus confirming EGR2 as a direct activator of the *ARF* promoter (Figure 4a,b, Figure S11).

Validation of the interaction between EGR2 and the *ARF* promoter was performed using chromatin immunoprecipitation (ChIP) on cross‐linked DNA from quiescent interleukin‐2 (IL‐2)‐dependent Kit225 human T‐lymphocytes, with low levels of *ARF* expression and Kit225 cells following IL‐2 activation which results in increased *ARF* expression (Gutierrez del Arroyo, 2007). Subsequently, ChIP was performed with polyclonal antibodies against EGR2 or E2F1, which acted as a positive control. Addition of IL‐2 to Kit225 cells resulted in significantly increased binding of E2F1 and EGR2 to the *ARF* promoter, demonstrating that EGR2 can be detected at the endogenous *ARF* promoter (Figure 4c).

In order to further explore the role of EGR2 in senescence, we introduced retroviral particles expressing EGR2 cDNA into normal human Hs68 diploid fibroblasts. In line with our previous observations that loss of EGR2 reverses senescence, stable overexpression of EGR2 was sufficient to induce proliferation arrest (Figure 4d,e). Interestingly, *p16*−/− Leiden cells and *p16*+/− Q cells also underwent proliferation arrest following overexpression of EGR2, indicating EGR2‐mediated up‐regulation of *ARF* is sufficient to induce senescence in the absence of *p16* (Figure 4d,e).

We next explored activation of the *p16* promoter and found that cells co‐transfected with one of each of the four members of the EGR2 family or E2F1, together with a pGL3 p16 construct displayed a significant increase in luciferase assay activation with the EGR2 or EGR4 expression vectors or E2F1 positive control, confirming EGR2 and EGR4 as direct activators of the *p16* promoter (Figure 4f). As EGR4 expression is not increased in DS compared to EP HMECs or HMFs ((GEO: GSE58035, Figure 3g), we suggest that EGR2 may be important for activation of the p16 promoter in epithelial and fibroblast senescence. Furthermore, ChIP performed in EP and DS HMFs revealed significantly increased binding of EGR2 to the *p16* promoter in DS HMFs, thus confirming that EGR2 can bind to the endogenous *p16* promoter (Figure 4g).

If EGR2 functions to activate the *p16* promoter and up‐regulate *p16* expression, we hypothesised that ablation of EGR2 in senescent cells would lead to a decrease in p16 levels. Subsequent investigation of DS HMFs transfected with an individual potent *EGR2* siRNA (‘DS + EGR2 *3* siRNA’) revealed a significant decrease in p16 protein levels compared to DS + siGLO HMFs (Figure 4h,i). Interestingly, the level of p16 in DS + EGR2 *3* siRNA HMFs was similar to DS + p16 + *p21* siRNA HMFs, indicating a down‐regulation of p16 in DS + EGR2 *3* siRNA HMFs comparable to reversed HMFs (Figure 4h,i). Using immunofluorescence staining and high‐content analysis, we further examined p16 and p21 on a cellular level and found a significant increase in the proportion of double negative (p16− p21−) ‘reversed’ cells in DS HMFs following EGR2 knockdown in combination with *p21* siRNA (‘DS + EGR2 + *p21* siRNA) compared to the DS + p21 siRNA HMFs, an increase similar to that seen in the reversed DS + p16 + p21 HMFs (Figure 4j,k). Taken together, these data indicate that EGR2 functions to transcriptionally up‐regulate *p16* and *ARF* expression in senescence which is sufficient to induce proliferation arrest, demonstrating that EGR2 acts as a novel transcriptional activator upstream of p16/pRB and ARF/p53/p21 pathways in senescence (Figure 4l).

## 3 DISCUSSION

Here, we show that DS can be transiently reversed in human fibroblasts using *p16* siRNA in combination with *p21* siRNA transfection, as characterised by the loss of a panel of senescence markers. It is important to note here that we have shown that siRNA mediated reversal of DS HMFs is transient, with population growth slowing and cells reverting to a senescence morphology by 7 days post‐transfection. Further investigation is required to assess the effect of long‐term, stable knockdown on DS cells, including the impact on DNA damage and telomeres. However, as previous work in our group demonstrated that *p16* siRNA knockdown can reverse DS HMECs, the discovery that *p16 *+ *p21* siRNA knockdown can transiently reverse DS HMFs provided a unique opportunity for uncovering novel senescence regulators in epithelial and fibroblast DS. Using siRNA screening, we identified novel regulators of senescence in HMECs and HMFs, including the transcription factor *EGR2*, extracellular matrix protein *FRAS1*, E3 ubiquitin ligase *RNF20* and calcium‐binding protein *S100A4*. Further investigation of the top hit, *EGR2*, revealed that *EGR2* ablation enables resumption of the cell cycle, reversed senescence‐associated morphologies and decreased expression and secretion of the SASP component, IL‐6. We demonstrate that EGR2 accumulates during in vitro senescence in DS HMECs, DS HMFs and OIS IMR90 lung fibroblasts. Furthermore, we re‐mined existing datasets to reveal an increase in EGR2 expression in RS HDFs, SIPS BJ fibroblasts, OIS WI38 fibroblasts and in human tissue during in vivo ageing. As such, we have identified EGR2 as a novel marker of senescence across multiple senescence models, including p16‐dependent epithelial DS, p16− and p21− dependent fibroblast DS, fibroblast RS, OIS and SIPS. Examination of genes differentially expressed in DS HMECs identified EGR2 binding sites in *p16* and nine siRNAs found to reverse DS HMEC and HMFs, including one top reversal hit in the DS HMFs, *S100A4*. Further investigation of the INK4/ARF locus revealed previously unreported EGR2 binding sites in all the *p16*, *p15* and *ARF* promoters. In support of this, we demonstrated that EGR2 activates the *p16* and *ARF* promoters and that EGR2 directly binds to both the *p16* and *ARF* promoters. Furthermore, stable EGR2 overexpression was sufficient to induce proliferation arrest in the presence or absence of p16. Lastly, we observed a decrease in p16 protein levels in DS HMFs following EGR2 knockdown and an increase in the p16− p21− double negative subpopulation in DS HMFs following EGR2 and p21 knockdown.

Mutations in EGR2 have been identified to lead to inherited peripheral neuropathies, including Charcot‐Marie‐Tooth Type 1 (Šafka Brožková et al., 2012), a demyelinating form associated with dysregulated Schwann cell proliferation and cell cycle exit (Atanasoski et al., 2006). Accumulating evidence indicates that EGR2, a transcription factor, plays the role of regulator in these processes (Decker, 2006; Topilko et al., 1994; Zorick et al., 1996) and has been shown to directly bind to the p21 promoter in myelinating rat sciatic nerve (Srinivasan et al., 2012). In addition, a role for EGR2 as a tumour suppressor has been implicated in many tumour cell types (Unoki & Nakamura, 2003), and elevated expression of EGR2 is a favourable prognostic factor in breast cancer (TCGA, 5‐year survival for high expressers = 84%; 5‐year survival for low expressers = 73%; *p* = 0.000073). Despite this, little attention has been paid to its role in senescence. In the present report, our findings indicate a functional role of EGR2 in transcriptional activation of *p16* and *ARF* in senescence.

Recently, EGR2 has been defined as a ‘pioneer’ transcription factor, potentially binding to the genome early on in the onset of senescence (Martínez‐Zamudio et al., 2020). Importantly, whilst our data demonstrate a role for EGR2 in regulation of senescence, transient EGR2 reversal in DS cells does not delineate between the activity of EGR2 in senescence onset or maintenance. Future studies using stable EGR2 knockdown prior to senescence entry should be performed in order to dissect the roles of EGR2 in the onset and/or maintenance of senescence.

## 4 CONCLUDING REMARKS

Our work adds to the growing list of pathways known to directly regulate senescence. This includes *p16* transcriptional repressors, such as homeobox protein HLX1 (Martin et al., 2013) and the N‐terminal fragment of the GLI2 transcription factor (Bishop et al., 2010), as well as *p16* transcriptional activators such as ETS1 (Ohtani et al., 2001) and homeodomain protein MEOX2 (Irelan et al., 2009). Importantly, we have demonstrated that EGR2 functions as a direct activator of p16/pRB and the ARF/p53/p21 pathways, thus controlling both axes of the senescence programme.

It is well established that expression of p16 increases with age in human tissues (Krishnamurthy et al., 2006), senescent cells accumulate in sites of age‐related diseases (Naylor et al., 2012), and selective clearance of p16‐positive senescent cells in mice has been shown to improve health and life span (Baker et al., 2011; 2016). As such, regulation of the p16/pRB and ARF/p53/p21 pathways by EGR2 in senescence may play an important role in ageing and age‐related diseases.

Furthermore, ten of these genes were identified as hits for senescence reversal in the DS HMEC screen, including *p16*, and nine of these were also identified as hits in the HMF siRNA screen, including the top hit *S100A4*, suggesting that EGR2 may act as a senescence regulator by activating the expression of these genes.

Interestingly, EGR2 as a transcription factor has the potential to regulate a network of genes in senescence, and nine hits which reversed DS HMECs and HMFs were identified to possess an EGR2 binding site, thus we hypothesise that EGR2 may potentially regulate the expression of these genes in senescence, although this has yet to be investigated further. Future exploration of the transcriptome regulated by EGR2 in senescence could provide new insights into regulation of the senescence programme and potentially identify essential senescence mediators, which could be exploited to eliminate senescent cells. As implications for senescence have been described in vivo for organismal ageing and age‐related diseases, furthering our understanding of this network in senescence could enable identification of therapeutic targets for treatment of ageing and age‐related diseases.

## 5 EXPERIMENTAL PROCEDURES

### 5.1 Cells and reagents

Normal finite life span HMECs and HMFs were obtained from reduction mammoplasty tissues of a 21‐year‐old individual, specimen 184 and 16‐year‐old individual, specimen 48, respectively, and were cultured as previously described (Garbe et al., 2009). Independent HMEC cultures were serially passaged from passage 6 (P6; early proliferating, EP) until p16‐dependent, p21‐independent stasis. Deeply senescent cultures underwent no further expansion upon at least two further weeks in culture (DS HMECs; Romanov et al., 2001; Garbe et al., 2009; Lowe et al., 2015), and independent HMF cultures were serially passaged from P4 until the population reached senescence at P29. DS HMFs underwent no further expansion upon at least three further weeks in culture (P29 + 3). Cells were cultured at 37°C in the presence of 5% CO_2_ and atmospheric O_2_. All cells were routinely tested for mycoplasma and shown to be negative.

*IMR90 ER*:*STOP* (vector) or *ER*:*RAS* (OIS) IMR90 foetal lung fibroblasts were produced as described in (Hari et al., 2019) and were a kind gift provided by Juan Carlos Acosta. These were maintained in DMEM supplemented with 10% FBS and 2 mM l‐glutamine.

U2OS cells, primary human fibroblast strain Hs68, and Kit225 T‐lymphocyte cell line were maintained as previously described (Gutierrez del Arroyo, 2007). Leiden and Q cells were maintained as previously described (Irelan et al., 2009).

### 5.2 siRNA transfections

The fluorescently labelled siRNA targeting cyclophilin B (siGLO) was selected as this did not influence the phenotype of either EP or DS cultures (Figure S12). HMECs were transfected with 60 nM siGLO siRNA (Dharmacon) or *p16* siRNA (Qiagen) in 384‐well plates using Dharmafect 3 (Dharmacon). HMFs were transfected with 30 nM siGLO siRNA or *p16* siRNA or *p21* siRNA (Dharmacon) in 384‐well plates or 6‐well plates using Dharmafect 2 (Dharmacon). DS + siGLO, DS + p16 siRNA, DS + p21 siRNA or DS + p16 + *p21* siRNA cells were harvested for RTqPCR, Western blotting or immunofluorescence as detailed below.

### 5.3 Immunofluorescence

Standard fixation with 3.7% paraformaldehyde, followed by 0.1% Triton X permeabilisation and blocking with 0.25% BSA was performed prior to antibody incubations. Primary antibodies used were mouseα*p16* JC8 (1:200), mouseα8‐oxoguanine (1:100, MAB3560 Millipore), rabbitα*p21* (1:1,000, 12D1 Cell Signalling), rabbitαEGR2 (1:250, H220 Santa Cruz), goatαIL‐6 (1:100, AB‐206‐NA R&D Systems), followed by donkeyαmouse AlexaFluor‐488 or goatαrabbit AlexaFluor‐546 (1:500, Invitrogen), DAPI and Cell Mask Deep Red (1:10,000, Invitrogen). For 5‐bromo‐2′‐deoxyuridine (BrdU) assays, cells were cultured in 5 µM for 16 h prior to fixation. An additional DNA denaturation step with 4 M HCl for 10 min was performed following permeabilisation, and a conjugated mouseαBrdU‐AlexaFluor‐488 antibody (1:100, B35130 Invitrogen) used. Images were collected at 10X using the IN Cell 1000 microscope (GE) and the Developer analysis software (GE) was used for image analysis as described previously (Bishop et al., 2010).

Please also see Appendix S1.

## CONFLICT OF INTEREST

The authors declare no competing interests.

## AUTHOR CONTRIBUTIONS

E.J.T., A.G.dA and C.L.B. conceptualised the sudy. E.J.T., A.G.dA, J.C.G., M.R.S. and C.L.B. involved in methodology. E.J.T., A.G.dA., B.K.H., R.W., R.L. and C.L.B. investigated the study. J.C.G., M.R.S. and J.K. involved in resources. E.J.T. and C.L.B. wrote the original draft. E.J.T., A.G.dA., B.K.H., R.W., J.C.G., M.R.S., J.K., R.L., M.P. and C.L.B. wrote, reviewed and edited the manuscript. M.P. and C.L.B. supervised the study.

## Supporting information

Supplementary Material

Table S1

## Data Availability

Data sharing is not applicable to this article as no new data were created or analysed in this study.
