# Supplementary material for: Early growth response 2 (EGR2) is a novel regulator of the senescence programme
Source: Aging Cell. 2021 Feb 6;20(3):e13318. doi: 10.1111/acel.13318 (PMC7963333; doi:10.1111/acel.13318)
Supplement: Supplementary file 1 — Supplementary Material [file ACEL-20-e13318-s001.docx]

**Experimental Procedures – Supporting Information**

**siRNA screening and Z score generation**

In the DS HMEC screen, *p16* siRNA was amongst the 28 driver siRNAs identified to reverse HMEC p16-dependent DS (Lowe *et al.*, 2015). However, in DS HMFs, *p16* siRNA alone was found to not be sufficient to reverse senescence. With this in mind, *p16* siRNA was not included as a target siRNA in the DS HMF screen, bringing the total number to 60 target siRNAs (27 drivers and 33 interactors).

**Z score** = (mean value of two independent experiments for experimental siRNA – mean value of two independent experiments for siGLO)/SD for siGLO of two independent experiments. For each of the parameters analysed, significance was defined as more than one Z score away from the siGLO mean in order to increase the window for hit detection and allow as many hits to be identified as possible. Z scores are presented as a heatmap.

**Immunoblotting and densitometry analysis**

Cell were lysed in RIPA buffer supplemented with 4% protease cocktail inhibitor (Roche) and protein concentration was determined using the Bio-Rad Protein Assay kit (Bio-Rad). Lysates were re-suspended in 6X Laemmli Sample Buffer (0.1M Tris pH6.8, 20% glycerol, 1% β-mercaptoethanol, 1% sodium dodecyl sulphate (SDS), 0.01% Bromophenol blue) and used for immunodetection. Primary antibodies used were mouseα*p16* JC8 (1:1,000), rabbitα*p21* (1:1,000 12D1, Cell Signalling), rabbitαlamin B1 (ab16048 Abcam, 1:1,000) and rabbitαGAPDH (1:2,000 ab9485 Abcam). Protein separation was achieved by SDS-PAGE on 10-12% polyacrylamide gels and proteins were subsequently transferred to Hybond nitrocellulose membrane (GE) using the Bio-Rad Mini-PROTEAN III system. Membranes were blocked in 5% Milk/PBS-Tween for 1hr before overnight incubation with primary antibody at 4°C with the exception of mouseα*p16,* which was used at room temperature for 2hr and rabbitαGAPDH at room temperature for 1hr. Following 3X PBS-T washes, membranes were incubated with an appropriate horseradish peroxidase (HRP)-conjugated secondary antibody for 1hr. Bands were then visualised using Enhanced-Chemiluminescence (ECL, GE). Densitometry analysis was conducted using ImageJ software. Density levels were corrected for protein loading and were expressed relative to the negative siRNA control.

**Quantitative RT-PCR (RTqPCR)**

Total RNA was isolated using Qiazol (Qiagen) according to the manufacturer’s protocol. One microgram of total RNA was reverse transcribed by the Superscript III Reverse Transcriptase (Thermo Fisher Scientific) following manufacturer’s protocol. RTqPCR reactions were performed with SYBR Green Master Mix (ABI) using a 7500 Fast Real-Time PCR System (Applied Biosystems). For siRNA knockdown experiments, RNA was extracted from DS HMFs five days post-transfection. *GAPDH* levels were quantified for each cDNA sample in separate RTqPCR reactions and were used as an endogenous control. Target gene-expression levels were quantified using target specific probes. Values were normalised to the internal *GAPDH* control and expressed relative to siGLO transfected control levels (100%). All RTqPCR reactions were run in duplicate for two independent samples.

**Enzyme-linked immunosorbent assay (ELISA)**

For IL-6 analysis by ELISA, equal volumes of conditioned medium were used and assay performed as per the manufacturer’s instructions (R&D Systems, Human Il-6 DuoSet ELISA DY206). Each sample was represented twice on the plate. The absorbance readings were taken at 450nm and 570nm using a CLARIOstar Plus multi-mode plate reader (BMG Labtech). Protein concentration was then estimated according to a calibration curve obtained from the absorbance values of a dilution series of the supplied standard protein control.

**Luciferase assays**

Luciferase assays were performed with pGL3-ARF-736 bp, pGL3-ARF-3.4 kb, and pGL3-p16 (generated by Eiji Hara), as previously described (Matheu, Klatt and Serrano, 2005; del Arroyo *et al.*, 2007). pbabepuroEGR2 was a gift from Novartis in San Diego. pB6CMV and pB6CMVEGR1/2/3/4 were a present from FJ Rauscher lab.

**Chromatin Immunoprecipitation (ChIP)**

ChIP analyses for the *ARF* promoter were performed with antibodies against EGR2 (C-14, sc-190) and E2F1 (H-137, sc-22820) using oligonucleotide primers specific for ARF (Forward: CCCTCGTGCTGATGCTACTG, Reverse: ACCTGGTCTTCTAGGAAGCGG), as previously described (del Arroyo *et al.*, 2007). ChIP analyses for the *p16* promoter were performed with the EGR2 antibody (H220, Santa Cruz) using the CUT&RUN assay kit (86652S, Cell Signaling Technology) according to manufacturer’s instructions and oligonucleotide primers specific for the INK4a ATG start site (Forward: CAGAGGGTGGGGCGGA, Reverse: CTCCATGCTGCTCCCCG).

**Database searches**

Protein interaction datasets were generated using the BioGRID bioinformatics database (<http://www.thebiogrid.org>). These interactions were then overlaid to generate a network requiring that each interactor generated a chain with at least two other drivers, revealing a total of 33 protein interactions. Using this method, only one protein interaction network was generated. We searched KEGG pathway (<http://www.genome.jp/kegg/pathway.html>) and PANTHER (<http://www.pantherdb.org>) databases to assign functional annotations to the 28 hits that strongly induced the reversal phenotype (‘drivers’) in the DS HMEC siRNA screen as well as the 14 hits in the DS HMF siRNA screen.

**Gene Expression Omnibus (GEO) dataset mining**

GEO datasets (GSE41714, GSE13330, GSE18876) available at ncbi.nlm.nih.gov/gds.

**Quantification and Statistical Analysis**

An un-paired, two-tailed t-test was performed to compare the means of two groups using the Microsoft Office Excel Analysis ToolPak (Microsoft, USA). A one-way analysis of variance (ANOVA) was used to analyse the differences between the means of three or more independent groups using Prism 7 (GraphPad Software Inc., USA). A two-way ANOVA was used to analyse the differences between multiple subgroups within multiple independent groups using Prism 7. Post-hoc statistical analysis was performed using either a Dunnett’s or Tukey’s multiple comparison test. The Dunnett’s test was used to compare every mean to a control mean, whereas the Tukey test was used to compare every mean with every other mean.

**Supplementary_Figure_1**


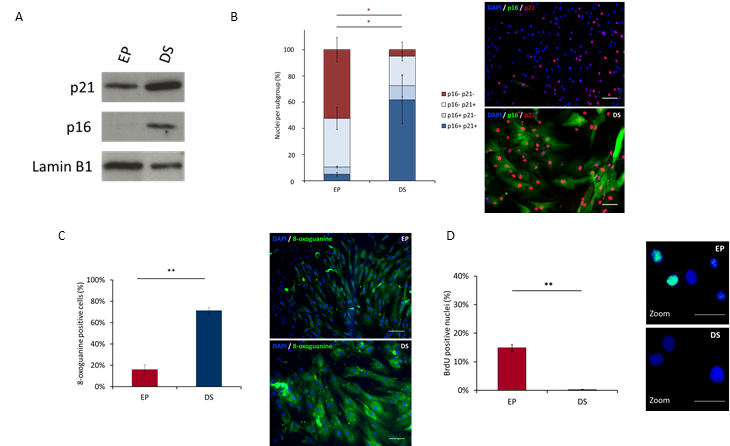


**Figure S1. Further validation of deep senescence with an extended panel of markers.**

**(A-D)** EP HMFs were seeded at 10,000 cells/cm^2^ and DS HMFs were seeded at 15,000 cells/cm^2^ in 96-well plate format or 384-well plate format and harvested after five days for western blot analysis (B) or immunofluorescence (D-E). **(A)** Western blot depicting p21, p16, and lamin B1 levels in EP and DS HMFs loaded by equal cell number. Lysates were probed for rabbit anti-p21 (12D1), mouse anti-p16 (JC8), and the rabbit anti-lamin B1 antibody. **(B)** EP and DS cells were stained with DAPI, mouse anti-p16 (JC8), rabbit anti-p21 (12D1), donkey anti-mouse Alexa Fluor 488, goat anti-rabbit Alexa Fluor 546, and nuclear intensities were quantitated. Nuclear intensity thresholds were established for p16 and p21 to define positive or negative nuclei. Nuclei were classified into four subgroups: p16 and p21 negative (p16- p21-); p16 negative and p21 positive (p16- p21+); p16 positive and p21 negative (p16+ p21-); and p16 and p21 positive (p16+ p21+). Bars denote mean percentage of nuclei per subgroup. Two-way ANOVA and Tukey’s test * p<0.05. Significance colours match nuclei subgroup. N=2 throughout. Error bars, SD of two independent experiments, each performed with three replicates. Representative immunofluorescence images of EP and DS fibroblasts. DAPI (blue), p16 (green), p21 (red). Size bar, 100μm. **(C)** EP and DS cells were stained with DAPI, mouse anti-8-oxoG, donkey anti-mouse Alexa Fluor 488, and 8-oxoG cellular density was quantitated. A cellular density threshold was established to define 8-oxoG positive or negative cells. Bars denote mean percentage of 8-oxoG positive cells. Un-paired two-tailed t-test ** p<0.01. N=2 throughout. Error bars=SD of two independent experiments, each performed with three replicates. Representative immunofluorescence images of EP and DS fibroblasts. DAPI (blue), 8-oxoG (green). Scale bar denotes 100μm. **(D)** EP and DS cells were stained with DAPI and mouse anti-BrdU Alexa Fluor 488. Using the secondary only control, a nuclear intensity threshold was established to define BrdU positive or negative nuclei. Bars denote mean percentage of BrdU positive nuclei. Un-paired two-tailed t-test ** p<0.01. N=2 throughout. Error bars, SD of two independent experiments, each performed with three replicates. Representative immunofluorescence images of EP and DS fibroblasts. DAPI (blue), BrdU (green). Digital zoom. Size bar, 50μm.

**Supplementary_Figure_2**

**
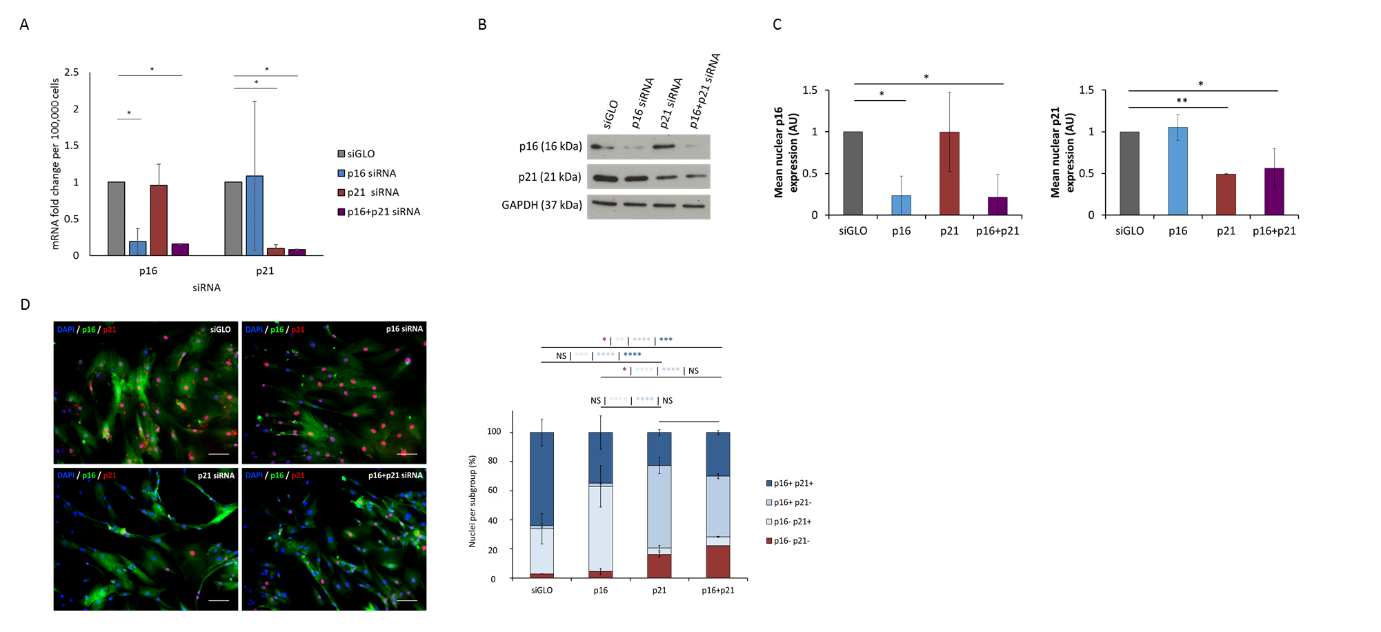
Figure S2. Further confirmation of reversal of deep senescence with an extended panel of markers.**

**(A-D)** DS HMFs were seeded at 15,000 cells/cm^2^ in 96-well plate format or 384-well plate format and forward transfected with 30nM control siRNA (siGLO), 30nM *p16* siRNA (p16), 30nM *p21* siRNA (p21), or 15nM *p16* siRNA together with 15nM *p21* siRNA (p16+p21) and harvested for RTqPCR at 72 hours post transfection (A) or at five days post transfection for western blot analysis (B-C) or immunofluorescence (D). **(A)** RTqPCR analysis of mRNA levels of *p16* and *p21* in DS HMFs following transfection with control siRNA (siGLO), *p16* siRNA (p16), *p21* siRNA (p21), or *p16* siRNA together with *p21* siRNA (p16+p21). * p<0.05, ** p<0.01. Error bars=SD from two independent experiments, each performed with two replicates. **(B-C)** Western blot depicting p16, p21, and GAPDH levels in DS HMFs following transfection with siGLO, *p16* siRNA, *p21* siRNA, and *p16* in combination with *p21* siRNA (p16+p21 siRNA). Lysates were probed for rabbit anti-p21 (12D1), mouse anti-p16 (JC8), and the rabbit anti-GAPDH antibody. Densitometry analysis of p16 and p21 levels in transfected DS HMFs. * p<0.05, ** p<0.01. Error bars, SD normalised to siGLO siRNA of two independent experiments. **(D)** Nuclear intensity thresholds were established for p16 and p21 to define positive or negative nuclei. Nuclei were classified into four subgroups: p16 and p21 positive (p16+ p21+); p16 positive and p21 negative (p16+ p21-); p16 negative and p21 positive (p16- p21+); and p16 and p21 negative (p16- p21-). Bars denote mean percentage of nuclei per subgroup. Two-way ANOVA and Tukey’s test * p<0.05, ** p<0.01, *** p<0.001, **** p<0.0001. NS=not significant. Significance colours match nuclei subgroup and are ordered left to right in the following order: p16- p21-; p16- p21+; p16+ p21-; p16+ p21+. N=2 throughout. Error bars, SD of two independent experiments, each performed with three replicates. Representative immunofluorescence images of siGLO, *p16*, *p21*, and *p16*+*p21* siRNA transfected DS HMFs. DAPI (blue), p16 (green), p21 (red). Size bar, 100μm.

**Supplementary_Figure_3**


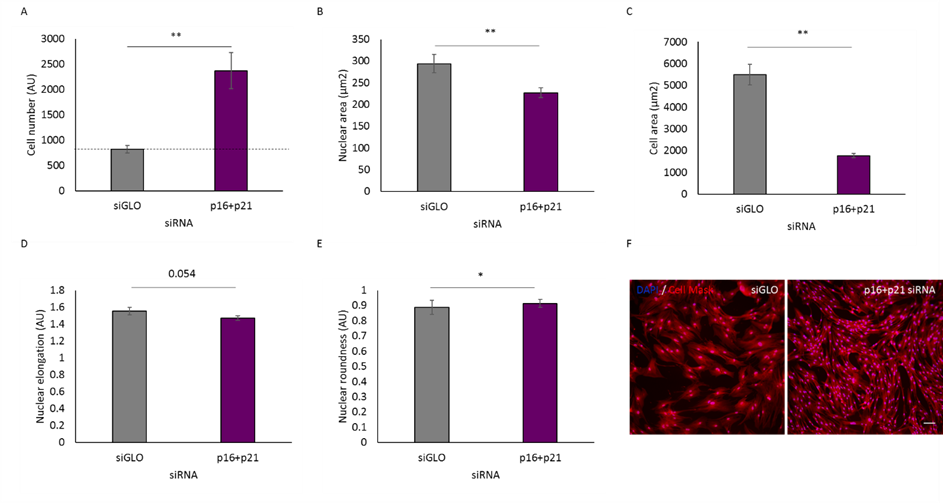


**Figure S3. Deep senescence is reversible in primary adult human dermal fibroblasts (HDFs).**

**(A-F)** DS HDFs were seeded at 10,000 cells/cm^2^ in 384-well plate format and forward transfected with 30nM siGLO (siGLO), 30nM *p16* siRNA (p16), 30nM *p21* siRNA (p21), or 15nM *p16* together with 15nM *p21* siRNA (p16+p21). After five days, cells were fixed, stained with DAPI and Cell Mask, imaged, and quantified. **(A)** Bar chart depicting mean cell number/well for each condition. Dashed line indicates original cell seeded number. ** p<0.01. Error bars, SD from two independent experiments, each performed with three replicates. **(B)** Bar chart depicting mean nuclear area (µm^2^) for each condition. ** p<0.01. Error bars, SD from two independent experiments, each performed with three replicates. **(C)** Bar chart depicting mean cell area (µm^2^) for each condition. ** p<0.01. Error bars, SD from two independent experiments, each performed with three replicates. **(D)** Bar chart depicting mean nuclear elongation (AU) for each condition. Error bars, SD from two independent experiments, each performed with three replicates. **(E)** Bar chart depicting mean nuclear roundness (AU) for each condition. * p<0.05. Error bars, SD from two independent experiments, each performed with three replicates. **(F)** Representative images of DS HDFs stained with DAPI (blue) and Cell Mask (red) following transfection with control siRNA (30nM siGLO) or 15nM *p16* siRNA together with 15nM *p21* siRNA (p16+p21). Size bar, 100µm.

**Supplementary_Figure_4**


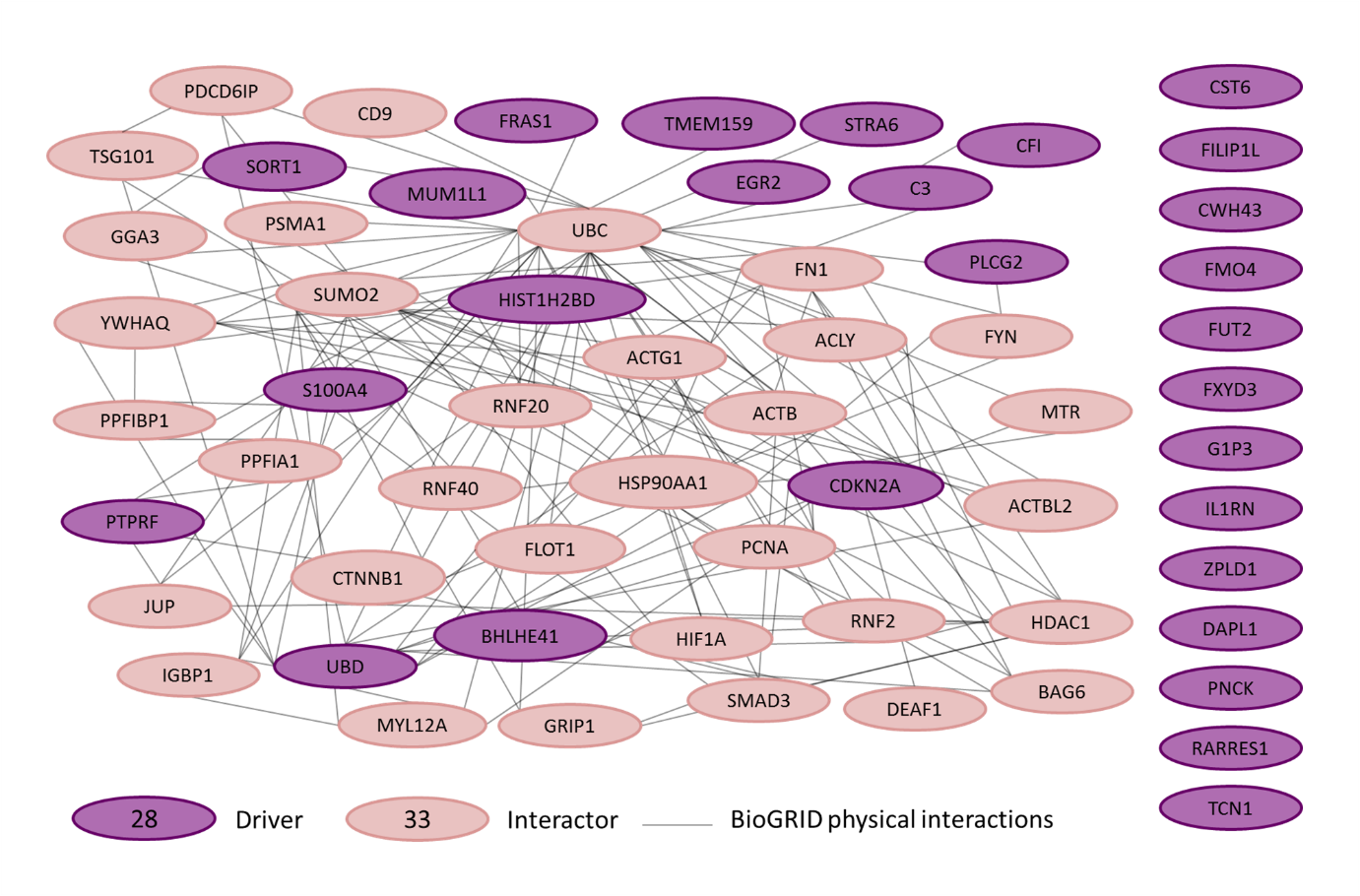


**Figure S4.** **Protein interaction network.** Interaction map for twenty-eight potential drivers of senescence (purple) identified in the previous DS HMEC siRNA screen and 33 interactors (pink) identified using bioinformatics to generate a chain with at least two other drivers. Lines represent physical interactions determined from the BioGRID database. Thirteen drivers were not found to be present in this network (listed on the right).

**Supplementary_Figure_5**


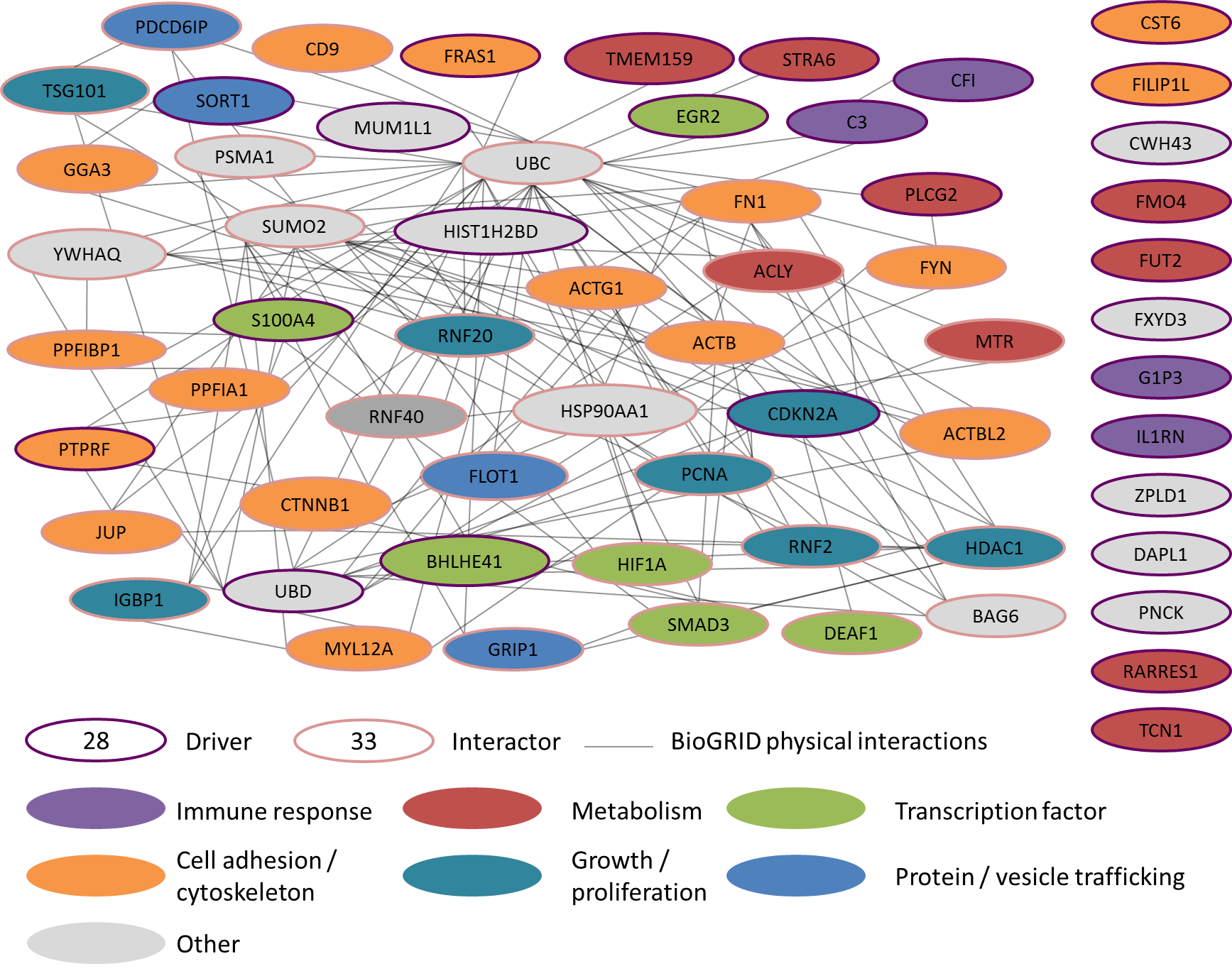


**Figure S5. Functional subgrouping of protein interaction network.** Using PANTHER, KEGG pathways, and GO bioinformatics tools, potential drivers (outlined in purple) and interactors (outlined in pink) were subgrouped into functional categories: immune response (purple), metabolism (red), transcription factor (green), cell adhesion/cytoskeleton (orange), growth/proliferation (teal), protein/vesicle trafficking (blue), other (grey). Lines represent BioGRID physical interactions.

**Supplementary_Figure_6**

**
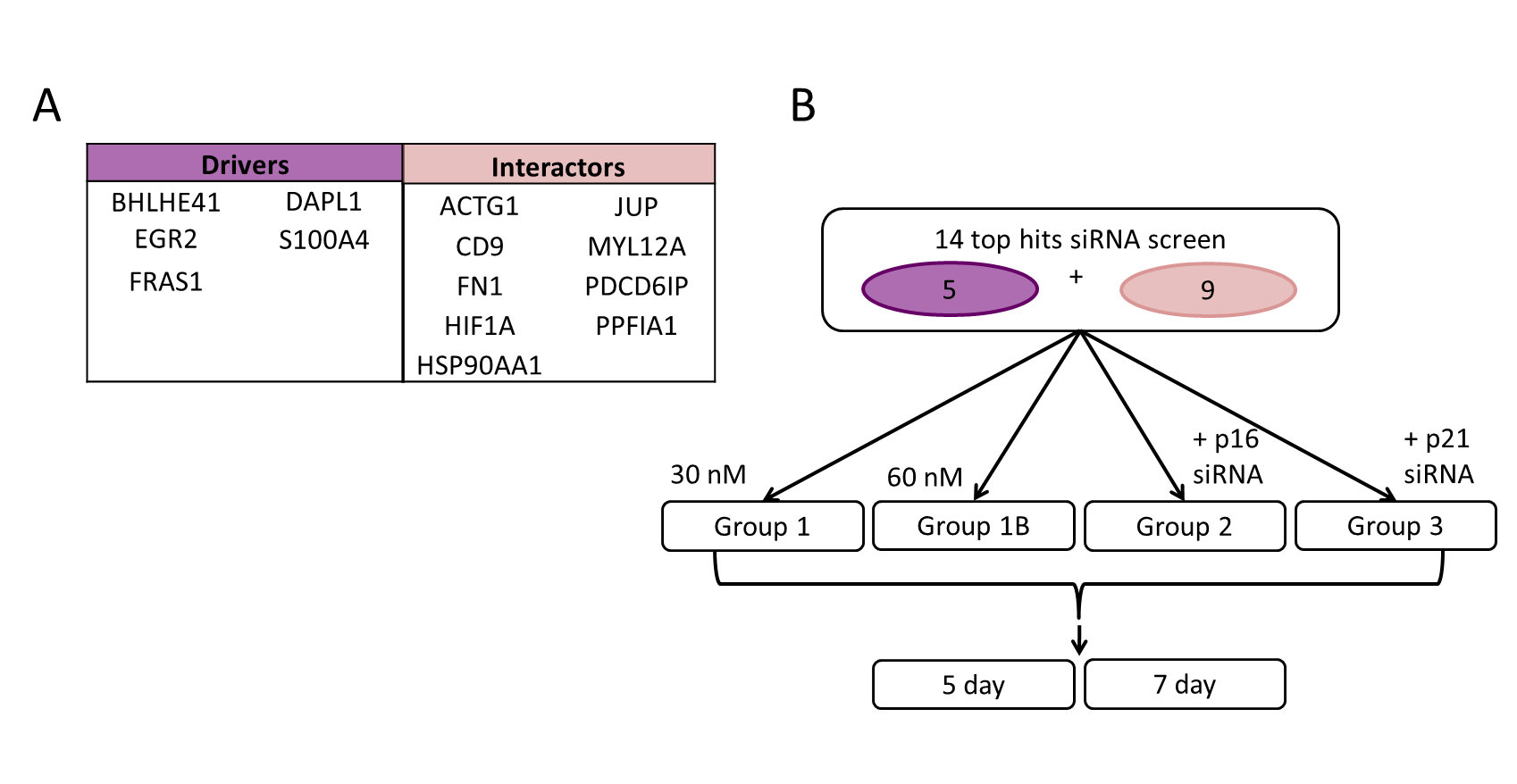
**

**Figure S6. Gene list of the 14 top hits and siRNA screening workflow for investigation of dose effect and time point extension**.

**(A)** Following the DS HMF screen, the 14 top hits were selected for further investigation: five drivers (purple), and nine interactors (pink). **(B)** Schematic illustrating the experimental design of the smaller siRNA screen investigating dose effect and time point extension in the top 14 hits. Using a previously optimised seeding density and transfection reagent dose, DS HMFs were forward transfected with the 14 siRNAs in four conditions: 30nM siRNA individually (Group 1); 60nM siRNA individually (Group 1B); 15nM siRNA in combination with 15nM siRNA (Group 2); and 15nM siRNA in combination with p21 siRNA (Group 3). After five or seven days, cells were fixed and stained with DAPI and Cell Mask, and quantified.

**Supplementary_Figure_7**
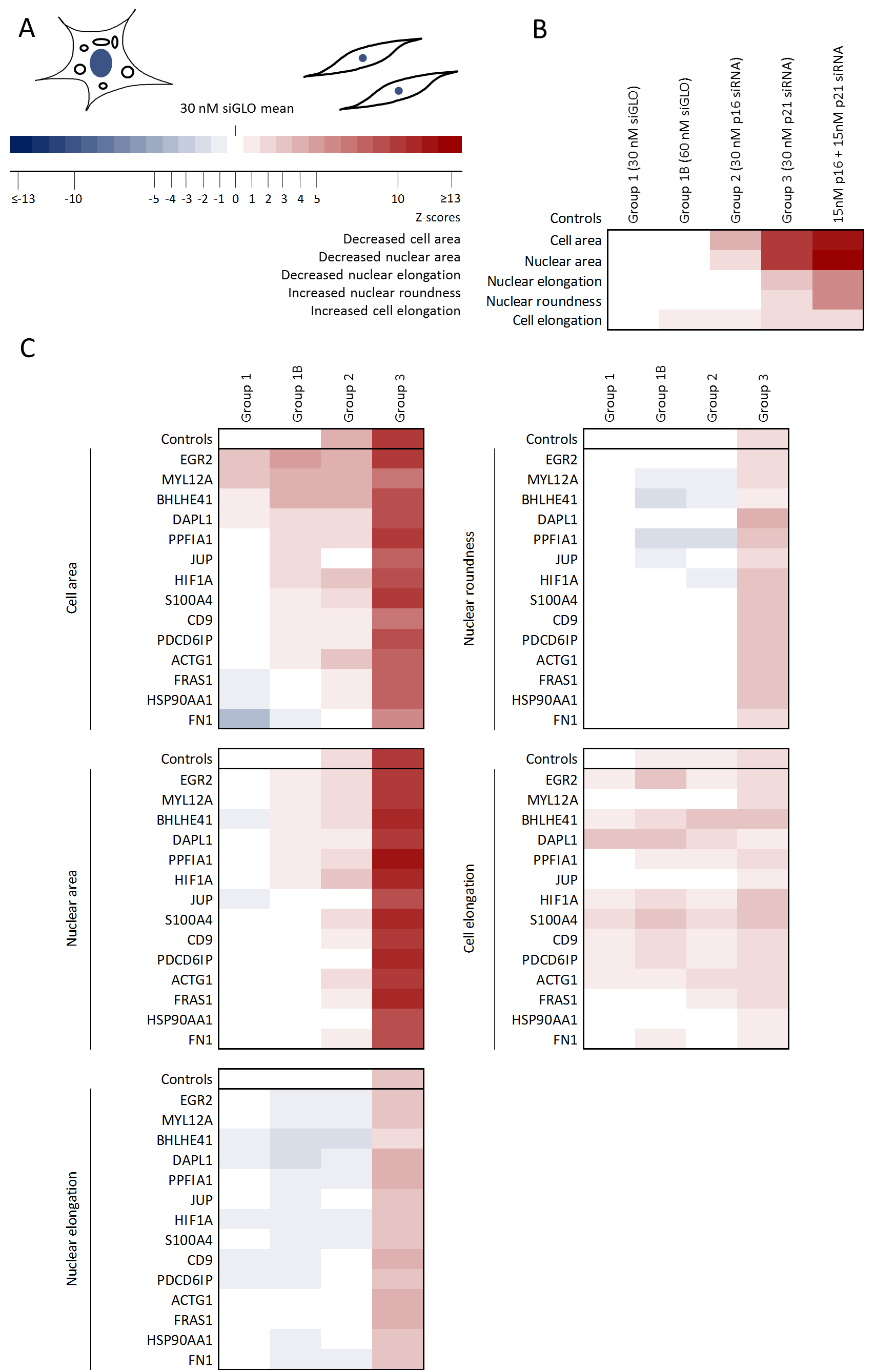


**Figure S7. Multi-parameter analysis of the top 14 siRNAs identified to reverse senescence in the DS HMFs at day five.**

Three independent siRNA screens, each in triplicate, were performed for the five day time point in DS HMFs. Cells were fixed, stained with DAPI and Cell Mask, imaged and nuclear and cellular morphologies quantified. Z scores were then generated. **(A)** Key. The colour saturation reflects the number of Z scores from the siGLO control mean. Scores highlighted in red denote a shift towards the reversed phenotype and blue denotes a shift away from the reversed phenotype. **(B)** Heatmap depicting significant changes in each of the panel of five morphological senescence-associated markers from the 30nM siGLO control mean (Group 1 control) for 60nM siGLO (Group 1B control), 30nM p16 siRNA (p16) (Group 2 control), 30nM p21 siRNA (p21) (Group 3 control), and 15nM p16 together with 15nM p21 siRNA (p16+p21) transfected DS HMFs. **(C)** Heatmap depicting significant changes in each of the panel of five morphological senescence-associated markers for the hit siRNAs selected from the previous screen in four different conditions: 30nM siRNA individually (Group 1); 60nM siRNA individually (Group 1B); 15nM siRNA in combination with 15nM siRNA (Group 2); and 15nM siRNA in combination with p21 siRNA (Group 3), compared to 30nM siGLO control mean.

**Supplementary_Figure_8**

**
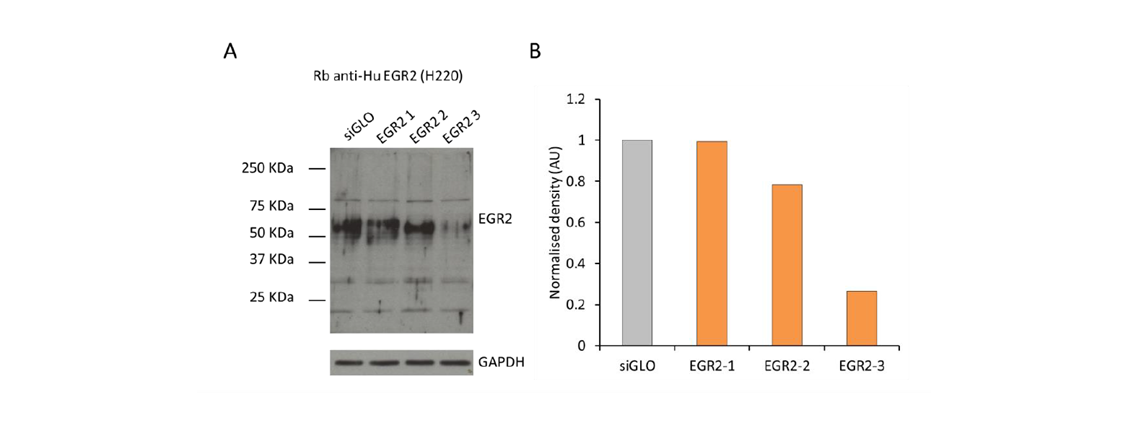
**

**Figure S8. Uncropped western blot of rabbit anti-human EGR2 (H220) antibody in DS HMFs transfected with individual EGR2 siRNAs. (A)** Representative western blot of DS HMFs transfected with 30nM siGLO (siGLO) or 30nM individual EGR2 siRNA (‘*EGR2 1*’, ‘*EGR2 2*’, *‘EGR2 3’* cell lysates probed for rabbit anti-human EGR2 (H220). **(B)** Densitometry analysis of EGR2 bands in rabbit anti-human EGR2 (H220) probed transfected DS HMFs. Analysis was performed using ImageJ software. Bars denote density levels normalised to GAPDH relative to the siGLO control.

**Supplementary_Figure_9**


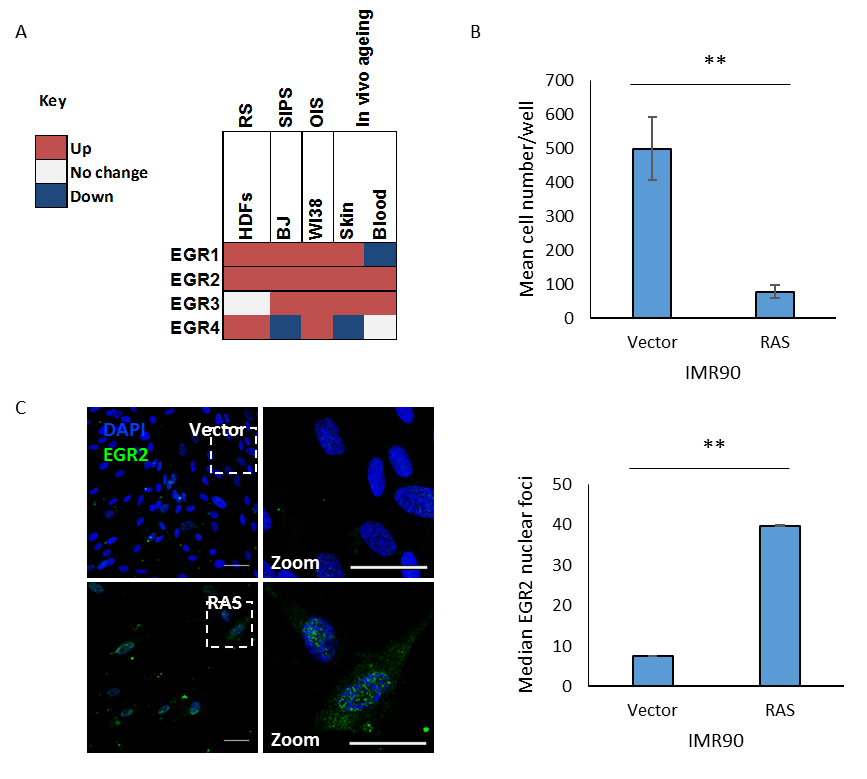


**Figure S9. EGR2 expression increases in multiple models of senescence *in vitro*, human ageing *in vivo*, and EGR2 protein levels increase in RAS oncogene-induced senescence (OIS) fibroblasts*.* (A)** EGR2 gene expression during *in vitro* senescence for replicatively senescent (RS) primary adult dermal fibroblasts (HDFs), bleomycin-induced stress-induced premature senescence (SIPS), RAS OIS in WI38 foetal lung fibroblasts, and *in vivo* ageing of human skin and blood. Red indicates an increase and blue indicates a decrease in EGR2 expression. **(B)** Barchart depicting mean cell number per well for vector and RAS OIS fibroblasts (RAS). ** p<0.01. Error bars, SD from three independent experiments, each performed with two replicates. **(C)** Representative immunofluorescence images of vector and RAS OIS fibroblasts stained with DAPI (blue) and EGR2 (green) at 5 days post-seeding. Barchart depicting median EGR2 nuclear foci in vector and RAS OIS fibroblasts (RAS). ** p<0.01. Error bars, SD from three independent experiments, each performed with two replicates.

**Supplementary_Figure_10**


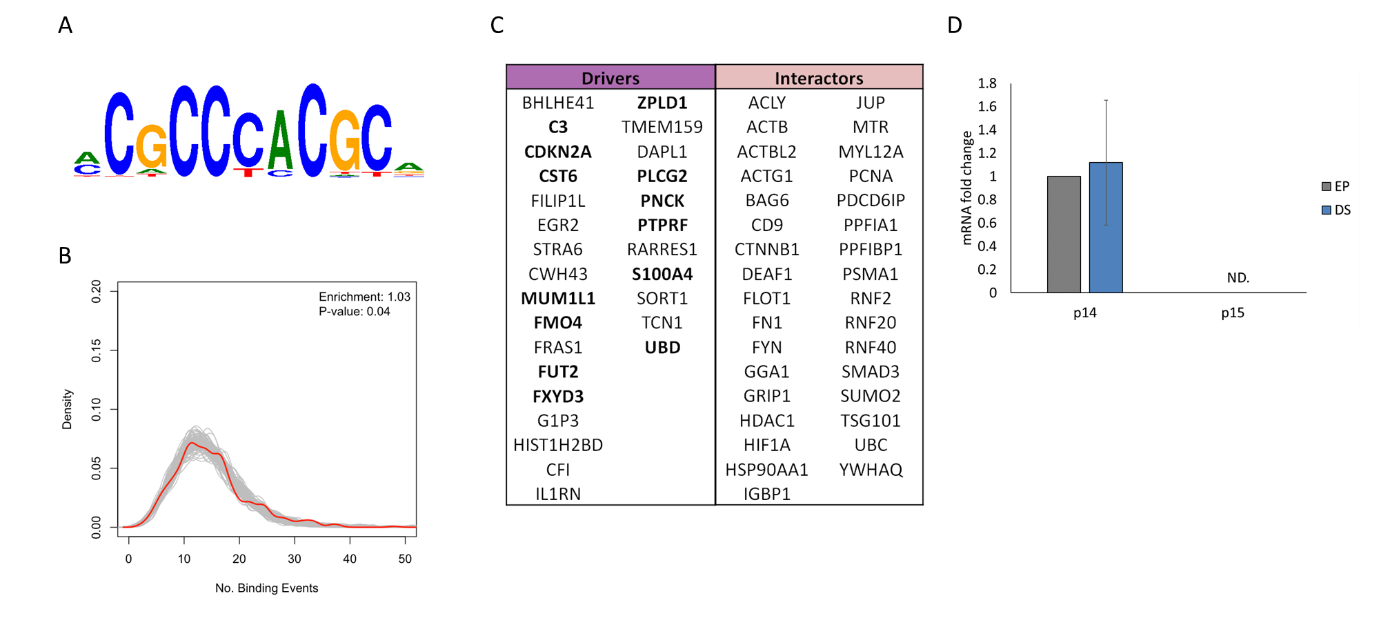


**Figure S10. Predicted EGR2 binding sites within siRNA screening hits and INK4/ARF locus.**

**(A)** EGR2 DNA binding motif (Jolma *et al.*, 2013; Mathelier *et al.*, 2016). **(B)** The EGR2 DNA binding motif was investigated in the promoters of genes up-regulated in HMEC senescence (red) relative to random sampling (grey). **(C)** The gene promoters predicted to contain EGR2 DNA binding motifs in the DS HMF siRNA screen are presented here in bold. **(D)** RTqPCR analysis of mRNA levels of *p14 (ARF)* and *p15* in EP and DS HMFs. Error bars, SD from two independent experiments, each performed with two replicates.

**Supplementary_Figure_11**

**
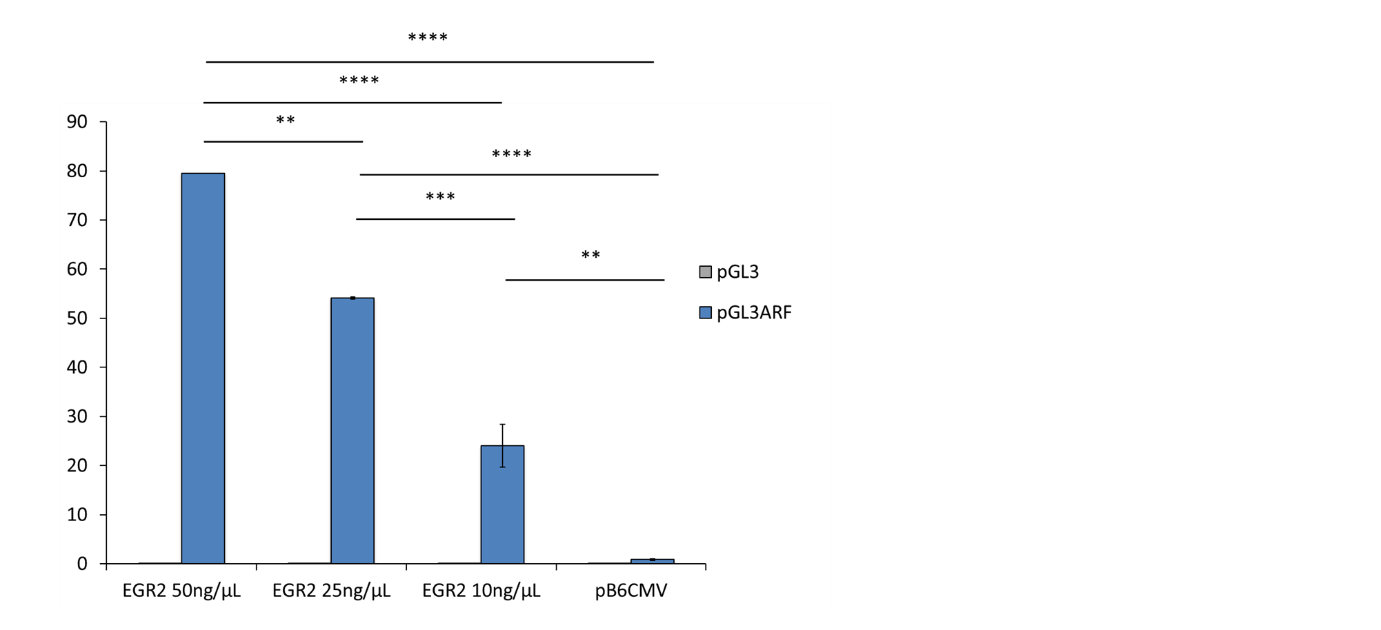
Figure S11. EGR2 expression vector titration results in ARF activation dose response.** Mean luciferase values for activation of pGL3 luciferase reporter constructs with and without the *ARF* promoter sequence (pGL3 or pGL3 ARF, respectively) following co-transfection of U2OS cells with titratable amounts of expression vector encoding EGR2 (50ng/µL, 25ng/µL, 10ng/µL, respectively) compared to pB6CMV vector backbone. ** p<0.01, *** p<0.001, **** p<0.0001. Error bars, SD from two experiments.

**Supplementary_Figure_12**
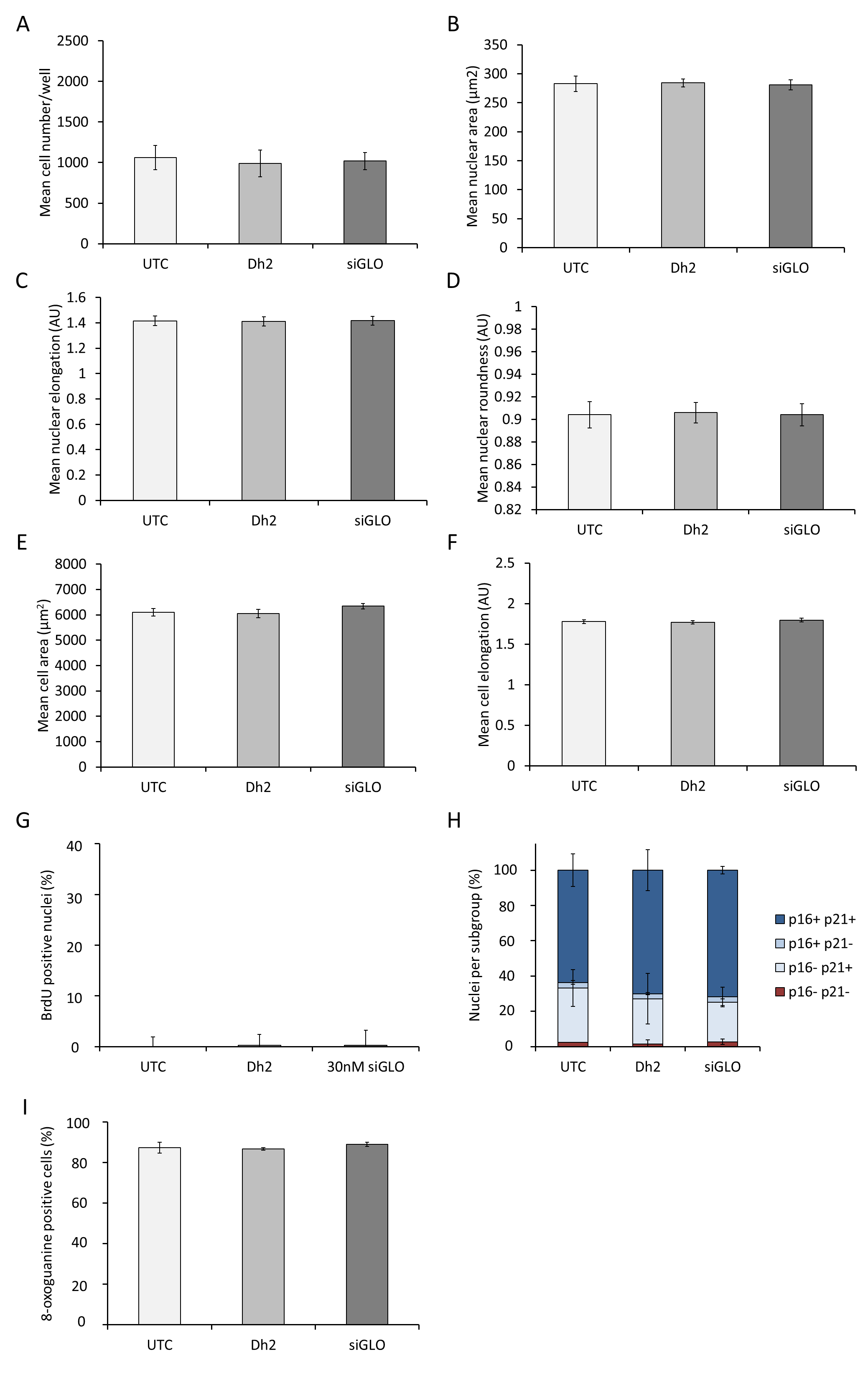


**Figure S12. Analysis of untransfected, transfection reagent only, and siGLO transfected controls.**

**(A-I)** DS HMFs were seeded at 15,000 cells/cm^2^ in 384-well plate format and were either untransfected (UTC) or forward transfected with either transfection reagent alone (Dh2), or with 30nM siGLO (siGLO). Cells were then fixed, stained with DAPI, mouse anti-BrdU Alexa Fluor 488, or mouse anti-p16 (JC8) and rabbit anti-p21 (12D1), or mouse anti-8-oxoguanine, and donkey anti-mouse Alexa Fluor 488 and goat anti-rabbit Alexa Fluor 546, and Cell Mask. Nuclear and cellular morphologies, BrdU nuclear intensity, p16 and p21 nuclear intensities, and 8-oxoguanine cellular density was quantitated. Using the secondary only control, a nuclear intensity threshold was established to define BrdU positive or negative nuclei. Nuclear intensity thresholds were established for p16 and p21 to define positive or negative nuclei. Nuclei were classified into four subgroups: p16 and p21 positive (p16+ p21+); p16 positive and p21 negative (p16+ p21-); p16 negative and p21 positive (p16- p21+); and p16 and p21 negative (p16- p21-). A cellular density threshold was established to define 8-oxoguanine positive or negative cells. **(A)** Bar chart depicting mean cell number/well. N=4. Error bars=SD of four independent experiments, each performed with three replicates. **(B)** Bar chart depicting mean nuclear area (μm^2^). N=4. Error bars=SD of four independent experiments, each performed with three replicates. **(C)** Bar chart depicting mean nuclear elongation (μm^2^). N=4. Error bars=SD of four independent experiments, each performed with three replicates. **(D)** Bar chart depicting mean nuclear roundness (AU). N=4. Error bars=SD of four independent experiments, each performed with three replicates. **(E)** Bar chart depicting mean cell area (μm^2^). N=4. Error bars=SD of four independent experiments, each performed with three replicates. **(F)** Bar chart depicting mean cell elongation (AU). N=4. Error bars=SD of four independent experiments, each performed with three replicates. **(G)** Bars denote mean percentage of BrdU positive nuclei. N=1. Error bars=SD of a single experiment containing three replicates. **(H)** Bars denote mean percentage of nuclei per subgroup. N=2. Error bars=SD of two independent experiments, each performed with three replicates. **(I)** Bars denote mean percentage of 8-oxoguanine positive nuclei. N=2. Error bars=SD of two independent experiments, each performed with three replicates.
