# Supplementary material for: Early growth response 2 (EGR2) is a novel regulator of the senescence programme
Source: Aging Cell. 2021 Feb 6;20(3):e13318. doi: 10.1111/acel.13318 (PMC7963333; doi:10.1111/acel.13318)
Supplement: Supplementary file 2 — Table S1 [file ACEL-20-e13318-s002.pdf]

Supplementary Table 1

Gene ID	Gene Symbol	Full Gene Name
56967	14orf132	chromosome 14 open reading frame 132
8310	ACOX3	acyl-Coenzyme A oxidase 3, pristanoyl
54	ACP5	acid phosphatase 5, tartrate resistant
59	ACTA2	actin, alpha 2, smooth muscle, aorta
8745	ADAM23	a disintegrin and metalloproteinase domain 23
283	ANG	angiogenin, ribonuclease, RNase A family, 5
288	ANK3	ankyrin 3, node of Ranvier (ankyrin G)
347	APOD	apolipoprotein D
84159	ARID5B	AT rich interactive domain 5B (MRF1-like)
91947	ARRDC4	arrestin domain containing 4
54469	AWP1	zinc finger, A20 domain containing 3
8424	BBOX1	butyrobetaine (gamma), 2-oxoglutarate dioxygenase (gamma-butyrobetaine hydroxylase) 1
7851	BENE	BENE
629	BF	B-factor, properdin
79365	BHLHB3	basic helix-loop-helix domain containing, class B, 3
684	BST2	bone marrow stromal cell antigen 2
64115	C10orf54	-
80183	C13orf18	chromosome 13 open reading frame 18
122525	C14orf28	chromosome 14 open reading frame 28
716	C1S	complement component 1, s subcomponent
718	C3	complement component 3
784	CACNB3	calcium channel, voltage-dependent, beta 3 subunit
147968	CAPN12	calpain 12
9236	CCPG1	cell cycle progression 1
1029	CDKN2A	cyclin-dependent kinase inhibitor 2A (melanoma, p16, inhibits CDK4)
1030	CDKN2B	cyclin-dependent kinase inhibitor 2B (p15, inhibits CDK4)
22802	CLCA4	chloride channel, calcium activated, family member 4
9976	CLECSF2	C-type lectin domain family 2, member B
25999	CLIPR-59	CLIPR-59
202333	CMYA5	cardiomyopathy associated 5
1308	COL17A1	collagen, type XVII, alpha 1
169044	COL22A1	collagen, type XXII, alpha 1
1291	COL6A1	collagen, type VI, alpha 1
1363	CPE	carboxypeptidase E
1368	CPM	carboxypeptidase M
1410	CRYAB	crystallin, alpha B
1474	CST6	cystatin E/M
1075	CTSC	cathepsin C
8722	CTSF	cathepsin F
1519	CTSO	cathepsin O
6376	CX3CL1	chemokine (C-X3-C motif) ligand 1
6372	CXCL6	chemokine (C-X-C motif) ligand 6 (granulocyte chemotactic protein 2)
1612	DAPK1	death-associated protein kinase 1
9201	DCAMKL1	doublecortin and CaM kinase-like 1
23586	DDX58	DEAD (Asp-Glu-Ala-Asp) box polypeptide 58
1672	DEFB1	defensin, beta 1

1675	DF	D component of complement (adipsin)
25891	DKFZP586H2123	DKFZP586H2123
11259	DOC1	DOC1
54800	DRE1	DRE1
1950	EGF	epidermal growth factor (beta-urogastrone)
1953	EGFL3	EGF-like-domain, multiple 3
1959	EGR2	early growth response 2 (Krox-20 homolog, Drosophila)
1999	ELF3	E74-like factor 3 (ets domain transcription factor, epithelial-specific)
8507	ENC1	ectodermal-neural cortex (with BTB-like domain)
956	ENTPD3	ectonucleoside triphosphate diphosphohydrolase 3
2043	EPHA4	EPH receptor A4
114907	FBXO32	F-box protein 32
55076	FLJ10134	transmembrane protein 45A
79656	FLJ11588	FLJ11588
64220	FLJ12541	stimulated by retinoic acid gene 6 homolog (mouse)
55601	FLJ20035	FLJ20035
54842	FLJ20160	FLJ20160
54863	FLJ20245	FLJ20245
79745	FLJ21069	restin-like 2
79600	FLJ21127	FLJ21127
80157	FLJ21511	FLJ21511
144568	FLJ25179	FLJ25179
158038	FLJ31810	leucine rich repeat neuronal 6C
153830	FLJ31951	FLJ31951
139221	FLJ33516	melanoma associated antigen (mutated) 1-like 1
389337	FLJ41603	FLJ41603
375287	FLJ45645	FLJ45645
2329	FMO4	flavin containing monooxygenase 4
80144	FRAS1	Fraser syndrome 1
64400	FTS	fused toes homolog (mouse)
2524	FUT2	fucosyltransferase 2 (secretor status included)
5349	FXYD3	FXYD domain containing ion transport regulator 3
2537	G1P3	interferon, alpha-inducible protein (clone IFI-6-16)
23710	GABARAPL1	GABA(A) receptor-associated protein like 1
352954	GATS	GATS
2634	GBP2	guanylate binding protein 2, interferon-inducible
9245	GCNT3	glucosaminyl (N-acetyl) transferase 3, mucin type
2852	GPR30	G protein-coupled receptor 30
84634	GPR54	G protein-coupled receptor 54
2877	GPX2	glutathione peroxidase 2 (gastrointestinal)
2896	GRN	granulin
26959	HBP1	HMG-box transcription factor 1
57801	Hes4	hairy and enhancer of split 4 (Drosophila)
3134	HLA-F	major histocompatibility complex, class I, F
3135	HLA-G	HLA-G histocompatibility antigen, class I, G
3229	HOXC13	homeo box C13
11145	HRASLS3	HRAS-like suppressor 3
54972	HSPA5BP1	heat shock 70kDa protein 5 (glucose-regulated protein, 78kDa) binding protein 1
3383	ICAM1	intercellular adhesion molecule 1 (CD54), human rhinovirus receptor
59082	ICEBERG	ICEBERG

3423	IDS	iduronate 2-sulfatase (Hunter syndrome)
3426	IF	I factor (complement)
3429	IFI27	interferon, alpha-inducible protein 27
64135	IFIH1	interferon induced with helicase C domain 1
3486	IGFBP3	insulin-like growth factor binding protein 3
3557	IL1RN	interleukin 1 receptor antagonist
286676	ILDR1	immunoglobulin-like domain containing receptor 1
79781	IQCA	IQ motif containing with AAA domain
3669	ISG20	interferon stimulated gene 20kDa
3017	IST1H2BD	histone 1, H2bd
3676	ITGA4	integrin, alpha 4 (antigen CD49D, alpha 4 subunit of VLA-4 receptor)
3708	ITPR1	inositol 1,4,5-triphosphate receptor, type 1
23043	KIAA0551	TRAF2 and NCK interacting kinase
23052	KIAA0830	-
11012	KLK11	kallikrein 11
26085	KLK13	kallikrein 13
25984	KRT23	keratin 23 (histone deacetylase inducible)
3920	LAMP2	lysosomal-associated membrane protein 2
3959	LGALS3BP	lectin, galactoside-binding, soluble, 3 binding protein
57146	LOC57146	LOC57146
92196	LOC92196	-
84171	LOXL4	lysyl oxidase-like 4
256691	MAMDC2	MAM domain containing 2
4126	MANBA	mannosidase, beta A, lysosomal
163071	MGC17986	zinc finger protein 114
79007	MGC3101	MGC3101
155382	MGC35352	Williams Beuren syndrome chromosome region 24
167359	MGC42105	MGC42105
83607	MGC4268	MGC4268
4256	MGP	matrix Gla protein
64386	MMP25	matrix metalloproteinase 25
4316	MMP7	matrix metalloproteinase 7 (matrilysin, uterine)
4582	MUC1	mucin 1, transmembrane
91663	MYADM	myeloid-associated differentiation marker
10529	NEBL	nebulette
4739	NEDD9	neural precursor cell expressed, developmentally down-regulated 9
4779	NFE2L1	nuclear factor (erythroid-derived 2)-like 1
4854	NOTCH3	Notch homolog 3 (Drosophila)
131368	OC131368	LOC131368
284207	OC284207	meteorin, glial cell differentiation regulator-like
284422	OC284422	-
342897	OC342897	LOC342897
349136	OC349136	LOC349136
401052	OC401052	LOC401052
169611	OLFML2A	olfactomedin-like 2A
5166	PDK4	pyruvate dehydrogenase kinase, isoenzyme 4
5336	PLCG2	phospholipase C, gamma 2 (phosphatidylinositol-specific)
57480	PLEKHG1	pleckstrin homology domain containing, family G (with RhoGef domain) member 1
139728	PNCK	pregnancy upregulated non-ubiquitously expressed CaM kinase

8611	PPAP2A	phosphatidic acid phosphatase type 2A
5792	PTPRF	protein tyrosine phosphatase, receptor type, F
51560	RAB6B	RAB6B, member RAS oncogene family
9052	RAI3	G protein-coupled receptor, family C, group 5, member A
399694	RaLP	RaLP
5918	RARRES1	retinoic acid receptor responder (tazarotene induced) 1
5920	RARRES3	retinoic acid receptor responder (tazarotene induced) 3
8786	RGS11	regulator of G-protein signalling 11
6038	RNASE4	ribonuclease, RNase A family, 4
79589	RNF128	ring finger protein 128
157869	RPESP	RPESP
10325	RRAGB	Ras-related GTP binding B
6275	S100A4	S100 calcium binding protein A4 (calcium protein, calvasculin, metastasin, murine placental homolog)
54809	SAMD9	sterile alpha motif domain containing 9
6340	SCNN1G	sodium channel, nonvoltage-gated 1, gamma
27111	SDCBP2	syndecan binding protein (syntenin) 2
6414	SEPP1	selenoprotein P, plasma, 1
5265	SERPINA1	serine (or cysteine) proteinase inhibitor, clade A (alpha-1 antiproteinase, antitrypsin), member 1
12	SERPINA3	serine (or cysteine) proteinase inhibitor, clade A (alpha-1 antiproteinase, antitrypsin), member 3
710	SERPING1	serine (or cysteine) proteinase inhibitor, clade G (C1 inhibitor), member 1, (angioedema, hereditary)
10610	SIAT7B	ST6 (alpha-N-acetyl-neuraminyl-2,3-beta-galactosyl-1,3)-N-acetylgalactosaminide alpha-2,6-sialyltransferase 2
84561	SLC12A8	solute carrier family 12 (potassium/chloride transporters), member 8
222962	SLC29A4	solute carrier family 29 (nucleoside transporters), member 4
154091	SLC2A12	solute carrier family 2 (facilitated glucose transporter), member 12
201266	SLC39A11	solute carrier family 39 (metal ion transporter), member 11
84189	SLITRK6	SLIT and NTRK-like family, member 6
150094	SNF1LK	SNF1-like kinase
6272	SORT1	sortilin 1
6775	STAT4	signal transducer and activator of transcription 4
10228	STX6	syntaxin 6
9066	SYT7	synaptotagmin VII
54843	SYTL2	synaptotagmin-like 2
6947	TCN1	transcobalamin I (vitamin B12 binding protein, R binder family)
29767	TMOD2	tropomodulin 2 (neuronal)
8793	TNFRSF10D	tumor necrosis factor receptor superfamily, member 10d, decoy with truncated death domain
94241	TP53INP1	tumor protein p53 inducible nuclear protein 1
23321	TRIM2	tripartite motif-containing 2
10537	UBD	ubiquitin D
9246	UBE2L6	ubiquitin-conjugating enzyme E2L 6
8408	ULK1	unc-51-like kinase 1 (C. elegans)
11045	UPK1A	uroplakin 1A
10451	VAV3	vav 3 oncogene
80232	WDR26	WD repeat domain 26
26118	WSB1	WD repeat and SOCS box-containing 1

7498	XDH	xanthine dehydrogenase
